# Tree diversity shapes soil bacterial community structure under low abiotic heterogeneity in the Atlantic forest

**DOI:** 10.1007/s00203-026-04776-y

**Published:** 2026-03-10

**Authors:** D. Tomachewski, R. F. Souza, D. R. Lammel, L. M. Schiebelbein, C. W. Galvão, M. F. Ribeiro, L. P. Karas, F. Galvão, V. A. Baura, M. C. Rillig, R. M. Etto

**Affiliations:** 1 Microbial Molecular Biology Laboratory, State University of Ponta Grossa, Ponta Grossa, Brazil; 2 Freie Universität Berlin, Institut für Biologie, D-14195 Berlin, Germany; 3 Berlin-Brandenburg Institute of Advanced Biodiversity Research (BBIB), D-14195 Berlin, Germany; 4 Forest Ecology Laboratory, Federal University of Paraná, Curitiba, Brazil; 5 Nucleus of Nitrogen Fixation, Federal University of Paraná, Curitiba, Brazil

**Keywords:** Soil microbial ecology, Tropical forest, Sustainable forestry, Tree-bacteria interactions, Ecosystem services

## Abstract

The Brazilian Atlantic Forest, a critical biodiversity hotspot, plays a vital role in climate regulation and water conservation. To explore the relationship between tree diversity and soil bacterial communities, soil samples were collected along an edapho-climatic gradient. We hypothesized that tropical forest soils, among Earth’s most complex biological environments, exhibit predictable microbial community assembly patterns driven by deterministic processes. Specifically: (i) tree composition influences bacterial communities more strongly than abiotic factors when environmental heterogeneity is low, following niche-based assembly rules; (ii) specific tree taxa function as keystone species, shaping bacterial communities through microhabitat modification. Our findings indicate that tree diversity had a greater influence on shaping bacterial communities than soil attributes. Of the 72 tree species identified, *Senegalia recurva*,* Araucaria angustifolia*,* Styrax acuminatus*,* Ilex paraguaiensis*,* Eugenia subterminalis*, and *Pisonia ambigua* were key drivers of soil bacterial diversity. Additionally, late-successional tree species that require high light were closely linked to predicted microbial cycling of carbon, nitrogen, sulfur, and phosphorus. These results offer a hypothesis-generating framework for evaluating species selection in Atlantic Forest restoration projects, suggesting that these species represent candidates whose effects can enhance soil health and ecosystem functionality in reforestation programs.

**Supplementary Information:**

The online version contains supplementary material available at 10.1007/s00203-026-04776-y.

## Introduction

Forest ecosystems cover approximately 30% of Earth’s land area (42 million km²) and store over 75% of terrestrial carbon, contributing to 50% of global net primary production (Terrer et al. 2021; Baldrian et al. 2023). However, climate change and land use pose critical uncertainties regarding the functioning and persistence of forest ecosystems (Willing et al. 2024). These soils harbor the largest proportion of global biodiversity among terrestrial environments, with complex multitrophic interactions that regulate essential ecosystem processes. The forest microbiome plays a key role in how forests respond to climatic stressors such as drought (Dudney et al. 2021), rising temperatures (Allsup et al. 2023), and elevated CO₂ (Pellitier et al. 2021). It is also crucial for forest recovery following fires (Pérez-Valera et al. 2020) and hurricanes (Liu et al. 2018b), influences soil methane production and consumption (Venturini et al. 2022), and affects the capacity of soils to sequester carbon (García-Palacios et al. 2021). The functioning of forests is driven by the activity of microbiomes, including fungi, bacteria, archaea, protists, and viruses (Baldrian et al. 2023). These microorganisms are especially vital in the topsoil, where they facilitate nutrient cycling, decompose organic matter, fix nitrogen, and maintain soil structure and fertility (Hättenschwiler et al. 2005; Hayat et al. 2010; Lammel et al. 2015; Tang et al. 2018; Lan et al. 2023; Baldrian et al. 2023). Therefore, the forest microbiome is a crucial component of soil, playing a key role in biogeochemical cycling and maintaining ecosystem health (Baldrian et al. 2023). However, there is now clear evidence that forest microbial communities are vulnerable to both climate and anthropogenic change, highlighting the need to deepen our understanding of the drivers shaping forest microbiomes in order to guide future management and conservation strategies (Willing et al. 2024).

The impact of soil characteristics on bacterial diversity is well-documented, with pH recognized as a major driver, while other factors, such as land use and other soil parameters, can also influence microbial diversity at smaller scales (Fierer et al. 2007; Bahram et al. 2018; Lammel et al. 2018; Sui et al. 2021). Environmental heterogeneity further shapes microbial communities by modulating the relative importance of deterministic and stochastic processes. At high heterogeneity, community structure is mainly determined by heterogeneous selection, promoting greater β-diversity. At intermediate heterogeneity, both heterogeneous and homogeneous selection act with similar strength, allowing stochastic processes to play a larger role, which reduces turnover and β-diversity. Under extremely homogeneous conditions, homogeneous selection dominates, filtering local communities and leading to low β-diversity (Huber et al. 2020). In forest ecosystems, plant diversity and topographic factors, along with soil properties, shape the assembly of soil microbial communities (Beugnon et al. 2021a; Bryant et al. 2008; Hui et al. 2017; Chodak et al. 2016). Recent studies have highlighted the significant role of tree root exudates in shaping the forest soil microbiome. The abundance and diversity of microorganisms in root exudates vary with tree diameter, indicating that trees influence soil microbial communities (Jing et al. 2023). Fine roots are active metabolic zones crucial for interactions with soil microbes (King et al. 2023). Chemical signals mediate plant-microbe communication, with tree roots secreting amino acids, organic acids, sugars, phenolic compounds, terpenoids, and sulfides that drive various biological processes (Pascale et al. 2020; Williams and de Vries 2020; Jing et al. 2023). These exudates influence soil microbial dynamics, enhance plant resilience to abiotic stress, and contribute to nutrient cycling and soil stability (Jing et al. 2023; Mommer et al. 2016; Wang et al. 2021). Roots release 10–50% of the carbon fixed by plants, playing a key role in the global carbon cycle (Nguyen 2003; Massalha et al. 2017; Prescott and Grayston 2023). While belowground traits like root exudates shape microbial interactions directly, aboveground plant structures such as the canopy also alter the soil microclimate, further influencing microbial dynamics (Yang et al. 2017).

Although some deterministic processes may be universal, their effects are largely shaped by the specific characteristics of boreal, temperate, and tropical forests and their microbiomes (Baldrian et al. 2023; Willing et al. 2024). Tropical forests have high structural complexity and greater tree species diversity, suggesting higher rhizosphere diversity and, consequently, more ecological niches (Trivedi et al. 2020; Baldrian et al. 2023). The Brazilian Atlantic Forest, with its rich biodiversity and home to approximately 2% of all global species, is composed of many subtypes of forests with unique fauna and flora, shaped by diverse soils, topography, and climatic conditions (Lima-Perim et al. 2016; Lladó et al. 2017; Souza et al. 2018; da Rosa et al. 2022). As a tropical forest, it helps mitigate global warming by sequestering large amounts of carbon dioxide, producing oxygen, regulating climate, supporting biodiversity, and protecting soil, all of which contribute to stabilizing Earth’s climate systems (Houghton 2005; Sun et al. 2022). In response to widespread degradation from agricultural expansion, Brazilian conservation laws have led to the creation of protected areas aimed at preserving biodiversity and restoring native Atlantic Forest ecosystems (Ferreira 1999). The Iguaçu National Park (INP) presents a unique opportunity for studying the native Brazilian Atlantic Forest, preserving four different pristine types of tropical forests that grow along a gradient of edapho-climatic conditions produced along an altitudinal gradient (Souza et al. 2017). Furthermore, the drivers of tree species growth in this park have been previously studied, with vegetation distributed across the gradient in response to deterministic factors such as variations in altitude, humidity, soil fertility, aluminum saturation, and clay content (Souza et al. 2018).

Given that tropical forests host a variety of trees that create distinct ecological niches for microorganisms, and considering the unique and well-characterized gradient of plants and edapho-climatic conditions in the INP (Souza et al. 2018), the objective of this study was to examine the influence of plant and soil parameters on soil bacterial communities. Since tree diversity has a positive effect on bacterial diversity and microbial physiological potential in forest soils (Beugnon et al. 2021a), we hypothesized that: (i) biotic drivers such as tree composition exert a stronger influence on microbial communities than abiotic factors when environmental heterogeneity is low; (ii) specific tree taxa function as keystone species that disproportionately shape bacterial community structure through ecological engineering mechanisms, including differential root exudation patterns and microhabitat modification.

## Materials and methods

### Area of study

The INP is a conservation area located in the state of Paraná, Brazil (Latitude: 25°03′07″ S and longitude: 53°37′57″ W). This park is the largest protected area of the Atlantic Forest in Brazil and provides a unique opportunity to investigate the drivers of microbial communities. The park’s elevation gradient results in distinct edapho-climatic conditions—such as differences in humidity, soil texture, nutrient availability, organic matter content, and pH—that drive changes in plant composition over short geographical distances. This rich and diverse setting enables the testing of hypotheses related to niche differentiation and keystone species theory, exploring how plant community composition influences microbial diversity and ecosystem functioning.

Within the altitudinal gradient, the park has a humid subtropical climate with a hot summer (Cfa) below 600 m and a humid subtropical climate with moderately warm summers (Cfb) above 600 m, where the mean temperature of the hottest month in the year does not exceed 22 °C. Furthermore, the average annual rainfall varies from 1831 mm to 1971 mm. The gradient also influences vegetation cover (Souza et al. 2018). According to the Brazilian Institute of Geography and Statistics (IBGE 2012) four main Atlantic Forest sub-types are identified in the area: Montane Mixed Ombrophylous Forest (MMOF), Submontane Semideciduous Seasonal Forest (SSSF), Alluvial Seasonal Semideciduous Forest (ASSF), and Montane Semideciduous Seasonal Forest (MSSF). The MMOF, SSSF, ASSF, and MSSF forests are characterized by diverse tree species, each forest type hosting a unique composition of over 50 to 300 different species (Souza et al. 2018).

The landscape of the INP encompasses the Iguaçu River hydrographic basin, with its altitudes varying from approximately 100 m to 800 m, from the southwest side to the north of the park. The park features two geomorphological compartments: the rugged, hilly terrain of the central north with dendritic drainage patterns, and the gently undulating hills of the south with rectangular drainage patterns. Moreover, five types of soils are present in the park: Rêndzic Chernossol, Gleisol, Litholic Neosol, Red Latosol, and Red Nitosol, with the latter two being the predominant types.

### Sampling

**Fig. 1:**
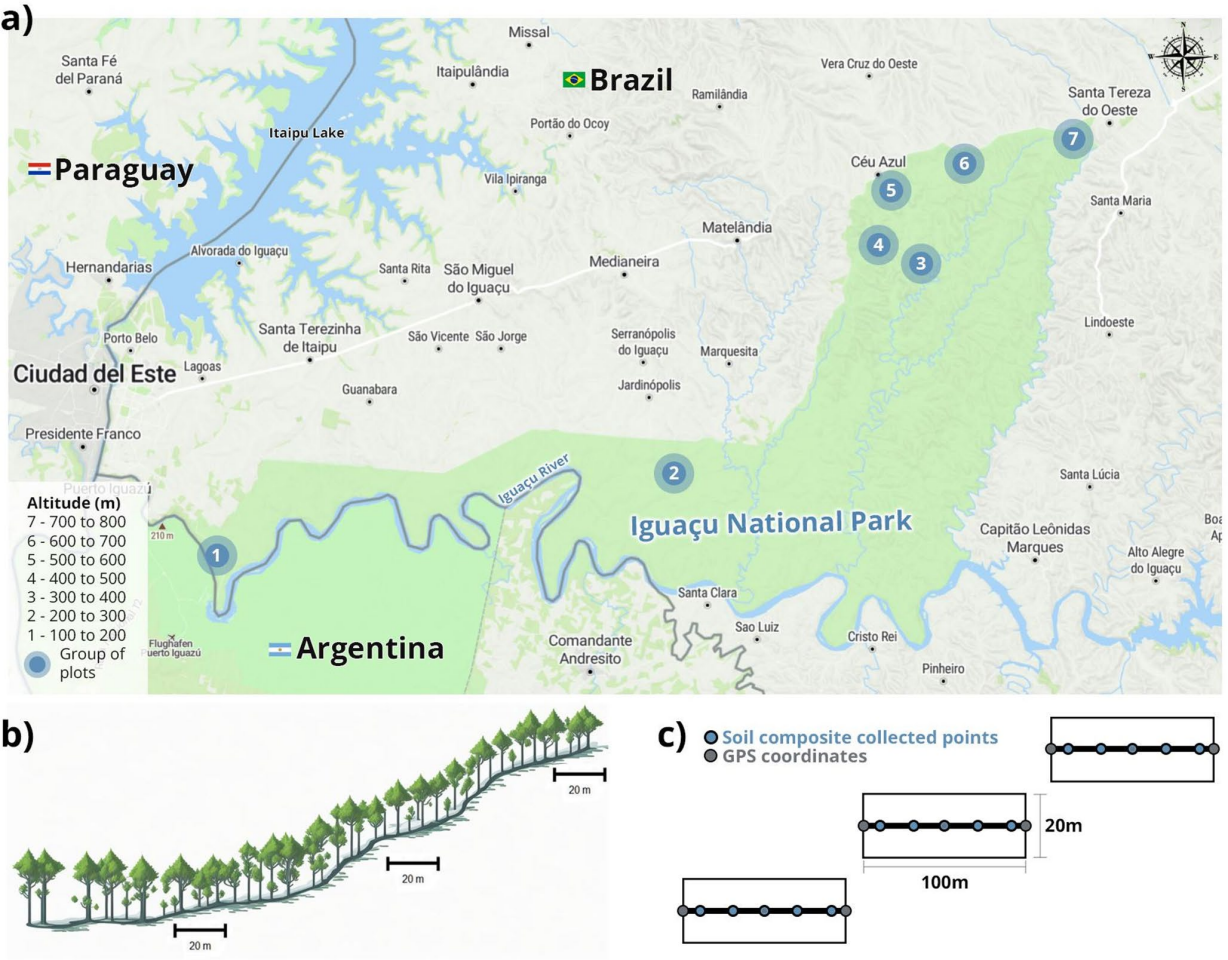
Panel illustrating the experimental design. The seven surveyed locations, each comprising three replicate plots, within Iguaçu National Park across varying altitude ranges (**a**). A schematic representation of plot distribution along the drainage slopes is shown (**b**), along with detailed layouts of survey locations within each replicate plot (**c**). The figure is adapted from MapTiler and Veloso et al. (1991)

The sampling in the INP was designed with fixed plots (Souza et al. 2017), positioned along a transect following an altitudinal gradient at seven different altitudes, from approximately 100 to 800 m of altitude (Fig. 1a). Each of the seven altitude groups consisted of three plots, positioned strategically along the drainage slopes (Fig. 1b), totaling 21 plots. The plots measured 20 × 100 m, constituting 2000 m^2^ in area (Fig. 1c). This design was implemented to ensure the sampling of the different levels of environmental gradients within the INP. The vegetation data and the soil chemical and physical properties were obtained from Souza et al. (2015, 2017, 2018). Plots dimensions were determined based on instructions provided by Augustynczik et al. (2013).

Given that vegetation varied according to the edapho-climatic conditions specific to each plot, and not solely due to altitude, we considered the 21 plots as the sampling units for this study (Souza et al. 2017). For each plot, a composite soil sample from 0 to 20 cm depth was taken by mixing five equal sub-samples along the central line of the plot. The survey line followed GPS coordinates to reference the plots (Fig. 1c). Five hundred grams of soil were collected for each plot and then used for chemical and molecular analysis.

### Attributes of the soil

 The following soil attributes were evaluated: pH; H⁺+Al⁺³; Al⁺³; Ca⁺²; Mg⁺²; P; K⁺; Sum of Bases (Ca⁺² + Mg⁺² + K⁺); Cation Exchange Capacity (SB + H⁺ + Al⁺³); Base Saturation (SB ÷ CEC × 100); Aluminum Saturation (Al⁺³ ÷ (SB + Al⁺³) × 100); percentage of sand, silt, and clay. Measurements of slope and soil drainage were defined according to Santos et al. (2007) (Table S1).

### DNA extraction and sequencing

The DNA from the composite soil samples from the 21 plots was extracted using the Power Lyzer Soil DNA Isolation Kit (MOBIO labs, Inc.) following the manufacturer’s instructions. The DNA concentration was measured with a Thermo Fisher Scientific, Nano-drop, and then the V4-V5 region of the 16 S rRNA gene was amplified using universal primers 515 F-Y and 926R (301 bp in final sequence output) (Quince et al. 2011; Parada et al. 2015) and KlenTaq Master Mix (Sigma). PCR was performed in triplicate for each sample as follows: 94 °C for 3 min; 18 cycles of 94 °C for 45 s, 50 °C for 30 s, and 68 °C for 60 s; followed by 72 °C for 10 min. The volumes were then pooled, quantified with a Thermo Fisher Scientific, Qubit, and purified with the PureLink PCR Purification Kit (Invitrogen). The samples were sequenced with the v2 Reagent Kit (500 PE cycles) on the MiSeq platform (Illumina, MiSeq) according to the manufacturer’s instructions. The sequencing data for this study are available at the National Center for Biotechnology Information (NCBI) Sequence Read Archive (SRA) under accession number PRJNA1179706 (URL: https://www.ncbi.nlm.nih.gov/sra/PRJNA1179706).

### Sequence analysis

The sequences were processed in R software (R Core Team 2021), and identified by barcodes through demultiplexing. DADA2 (Callahan et al. 2016) software was used to preprocess and generate the Amplicon Sequence Variants (ASVs), filtering out calibration quality control sequences, chimeras, and noise. A final matrix of 21 libraries and 64,762 ASVs was obtained. The libraries were rarefied to the lowest library’s sequence count of 60,902 reads using the SRS package in R (Heidrich et al. 2021).

To reduce noise and maintain diversity, only 3,886 ASVs with at least 0.005% (Bokulich et al. 2013) of the total sequences were retained. The remaining matrix underwent taxonomic identification using the SILVA database (Quast et al. 2012). Mitochondrial and chloroplast ASVs were checked for removal from the analysis, but none were found after the 0.005% cutoff. The final prokaryotic matrix contained 3,886 ASVs and was separated into matrices for bacteria (3420 ASVs), and unclassified taxa were excluded (466 ASVs). Furthermore, the bacterial matrix was also used to create a generalized matrix of bacteria at the phylum taxonomic level.

### Statistical analysis

**Fig. 2:**
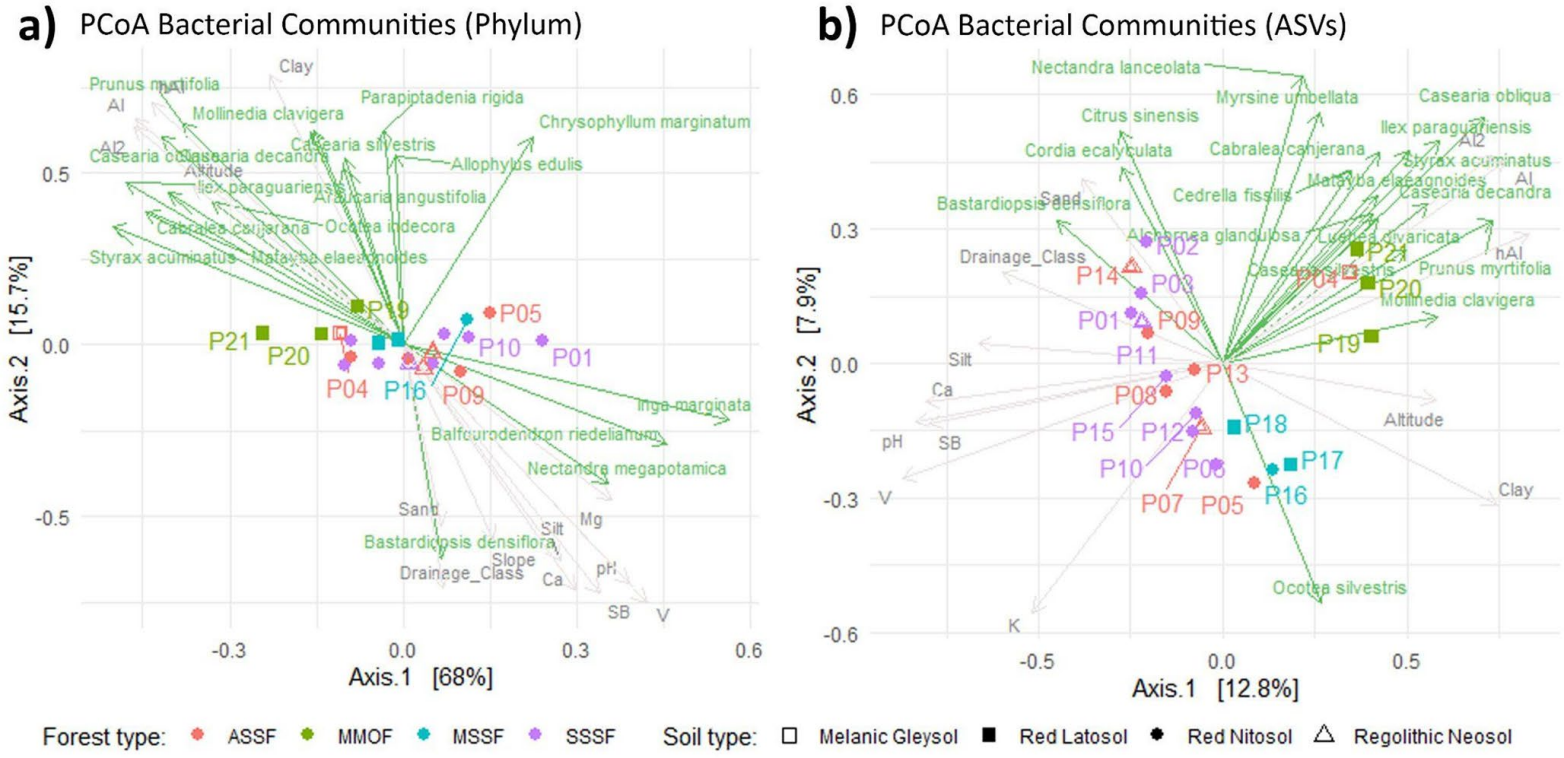
Influence of tree species and physical/chemical attributes on the spatial structure of bacterial communities. The PCoA of bacterial communities at the Phylum level (**a**) and at the ASV level (**b**). Physical attributes from Table S1 in gray, and tree species (Table S2) in light green, were added to the PCoA using the *envfit* function from the vegan R package with 9999 permutations. In the forest types, the acronyms mean: *ASSF* alluvial seasonal semideciduous forest, *MMOF* montane mixed ombrophylous forest, *MSSF* montane semideciduous seasonal forest, SSSF submontane semideciduous seasonal forest (Souza et al. 2018)

The statistical analyses were conducted using R software (R Core Team 2021). To analyze the variance explained in our bacterial matrix, we used the variation partitioning analysis from the Vegan package (Oksanen et al. 2001). The model used to perform the test employed a set of variables (soil physical and chemical properties, and vegetation) first selected from a principal component analysis (PCA) and principal coordinate analysis (PCoA) (Fig. 2 and Fig S1), then tested in all possible combinations that did not present collinearity or other errors in the *varpart* function. The combination that presented the lowest residual was selected to compose the final model. A redundancy analysis was performed using the function rda to test the significance of each explained component. Moreover, the variance explanation was also calculated with *Random Forest* (Breiman et al. 2002). Both analyses mentioned above were performed using the ASVs matrix, and the ASVs matrix generalized to the phylum taxonomic level.

A probabilistic species co-occurrence analysis of ASVs and trees was performed using the *cooccur* function from the cooccur package (Griffith et al. 2016). Only significant (*p* < *0.05*) and positive co-occurrences between ASV and tree were retained in order to generate the network plot.

The *indval* function from the *labdsv* package was used to identify indicator species (ASVs) for each tree in the INP. The vegetation matrix of tree species was transformed to presence and absence, characterizing two groups: 0 and 1, for which ASVs were calculated to be the indicators of such groups. Only the significant (*p* < *0.05*) ASV indicators of the presence of each tree were used to plot the network.

Correlations involving different groups (ASVs and tree species) were computed pairwise using Spearman correlation. We considered a correlation to be robust if Spearman’s correlation coefficient was greater than 0.4 and *p* < 0.05. Furthermore, each correlation was subjected to a permutation test with 1,000 permutations; the p-value was then corrected and a new cutoff of *p* < 0.05 was used. These thresholds are both mathematically and biologically significant: variables with high correlations often exhibit multicollinearity, indicating a strong link between them, and focusing on organisms that strongly co-occur increases the likelihood that they interact within the food web.

To create the network plots (correlation, co-occurrence, and species indicators), data tables from R Studio were exported to Gephi software (Bastian et al. 2009). In Gephi, Force Atlas was used to organize the nodes with a repulsion force of 15,000 and other parameters set to their standard values. Node sizes were set by ranking from one to four. To color the nodes and edges in the network and thereby visualize distinct clusters, a statistical community detection algorithm based on Modularity Class (Blondel et al. 2008) was applied.

 To assess the heterogeneity of microbial community composition across groups of plots in each altitude, we applied a multivariate dispersion analysis following Anderson (2006). First, all numerical variables from the sample metadata (Table S1) were standardized (mean = 0, standard deviation = 1) to avoid differences in scale among variables. Samples were then grouped according to altitude, which was treated as a categorical factor. We computed a Euclidean distance matrix based on the standardized data and used the betadisper function in the vegan package (Oksanen et al. 2001) to estimate within-group dispersion, expressed as the average distance of samples to their group centroid in multivariate space. The squared distances were used as a proxy for heterogeneity (analogous to I^2^ in meta-analysis), allowing comparison of variability among altitude groups. Environmental heterogeneity was additionally quantified as the mean Euclidean distance of samples to their altitude-group centroid using multivariate dispersion analysis (betadisper), a standard measure of within-group multivariate variability, corroborating to the multivariate dispersion analysis (Table S4).

 The PICRUSt2 (Phylogenetic Investigation of Communities by Reconstruction of Unobserved States) tool (Douglas et al. 2019) was used to perform the functional metagenomic prediction of ASVs. The metagenome functional potential was predicted using enzyme classification (EC) numbers. The relative abundance of EC numbers relevant to the metagenome function was identified through the MetaCyc database (Caspi et al. 2019). Furthermore, correlational data were also used to count the ASVs correlated with trees in each plot and in each different ecological group (Table S3 and Fig. 5). The data generated by the PICRUSt2 package and the *pred_metagenome_unstrat* table were normalized. Data were analyzed using the Z-Score function of the stats library from the *scipy* package (Virtanen et al. 2020). Enzyme functions were predicted from 16 S rRNA gene sequencing data using the PICRUSt2 pipeline. The generated EC (Enzyme Commission) number abundance table was filtered to include only enzymes relevant to the biogeochemical cycles of interest. For visualization in the heatmap, abundance values were normalized by calculating the Z-score for each enzyme (row) individually. This method standardizes the abundance of each enzyme around a mean of zero and a standard deviation of one among the samples. This transformation allows us to effectively identify in which samples a given enzyme is relatively over- or under-expressed relative to its own mean, facilitating visual comparison of patterns between enzymes with different absolute abundance scales. The Z-score represents the distribution of a numerical measure of the ratio of a value to the mean (Choi et al. 2018). The normalization method was used to calculate the relative abundance of each module (Zhang et al. 2022a, b).

## Results

### Determination of the main driver of the bacterial diversity in Atlantic forest soil

**Fig. 3:**
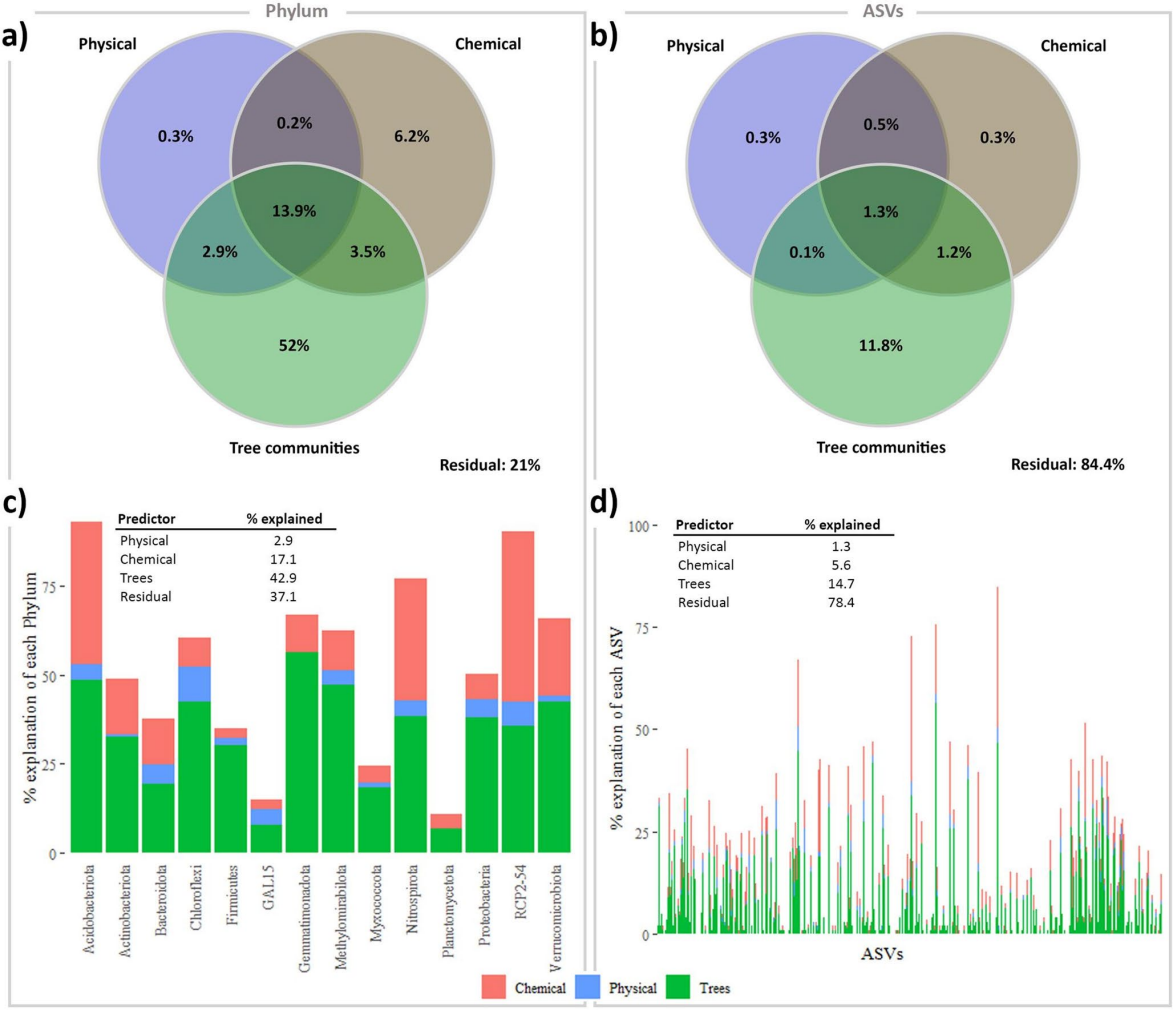
Bacterial diversity is more influenced by trees than by physical and chemical attributes. The Venn diagram based on *varpart* analysis, shows the bacterial community variance explained at the phylum level (**a**) and at the ASV level (**b**). The graph shows the variance explained by *Random Forest* analysis of each phylum of the bacterial community (**c**) and of each of the 3420 ASVs of the bacterial community (**d**)

Results indicated that bacterial diversity varied across the vegetation gradient, as demonstrated by beta-diversity represented by PCoA based on Bray-Curtis distance at both the bacterial phylum and ASV levels. Despite the park’s diverse soil types, the gradient was primarily influenced by tree composition (Fig. 2). To further investigate the effects of different parameters, including plant cover and soil chemical and physical attributes, variation partitioning and Random Forest analyses were conducted (Fig. 3).

 The *varpart* analysis (Fig. 3a and b), was based on PCAs and PCoAs performed to help create the model to be tested (Fig. 2 and Fig S1). From the PCA analysis based on soil attributes (Table S1), the physical attributes: Altitude, Slope, Soil type, Sand (%), Silt (%), and Clay (%); and chemical attributes: pH, Ca⁺², H^+^+Al, Al⁺³, SB, V, and Al (%) presented the greatest contribution to the first axis (38.8%). From the PCoA analysis based on 72 tree species (Table S2), 25 tree species presented the greatest contribution to the first axis (Fig. 2a and b).

After the exclusion of redundant attributes, all other attributes were submitted to an R script to test all combinations in *varpart* analysis. The best model for the phylum level selected the following: Altitude, Sand (%), Ca⁺², and SB as the main physical and chemical attributes, and from all the PCoA-selected trees, *Balfourodendron riedelianum*,* Bastardiopsis densiflora*,* Casearia decandra*,* Casearia silvestris*,* Inga marginata*,* Nectandra lanceolata*,* Nectandra megapotamica*,* Ocotea indecora*, and *Parapiptadenia rigida* were excluded, and *Chrysophyllum marginatum*,* Lonchocarpus campestris*,* Pisonia ambigua*, and *Trichilia claussenii* were added to compose the model. The best model for ASVs selected the following: Altitude, Sand (%), and Ca⁺² as the main physical and chemical attributes, and from all the PCoA-selected trees, the species *Alchornea glandulosa*,* Bastardiopsis densiflora*,* Cabralea canjerana*,* Casearia decandra*,* Casearia silvestris*,* Cedrella fissilis*,* Citrus sinensis*,* Luehea divaricata*,* Myrsine umbellata*,* Nectandra lanceolata*,* Ocotea silvestris* were excluded, and *Allophylus edulis*,* Chrysophyllum marginatum*,* Lonchocarpus campestris*,* Pisonia ambigua*, and *Trichilia claussenii* were added to compose the model.

The analysis of bacterial community variance revealed that tree species are the primary drivers, explaining 52% of the variance at the phylum level and 11.8% at the ASV level. Physical and chemical attribute effects accounted for 6.2% at the phylum level and 0.3% at the ASV level. When all three factors are considered together, they explain 13.9% of the variance at the phylum level and 1.3% at the ASV level, leaving a residual of 21% and 84.4%, respectively (Fig. 3a and b).

Random Forest analysis corroborated these findings, showing that trees are the dominant predictors of variance for both phylum and ASV levels. At the phylum level, trees explained 42.9%, chemical attributes 17.1%, and physical attributes 2.9% of the variance. At the ASV level, trees accounted for 14.7%, chemical attributes 5.6%, and physical attributes 1.3% (Fig. 3c and d).

The interspecific relationship among phyla, Amplicon Sequence Variants (ASV), tree species, and the physical and chemical attributes of the sampled locations showed that the tree community was dominant among the measured factors in shaping the bacterial community.

### Contribution of tree species to the soil bacterial diversity in the Atlantic forest

The analysis of species co-occurrence patterns in ecological communities utilizes a probabilistic model to determine whether pairs of species are found together more or less frequently than expected by chance. This assessment can indicate potential ecological interactions, such as competition or mutualism (Gotelli and McCabe 2002). The co-occurrences of each ASV with the trees, allow us to observe the locations and frequency of these interactions (Fig S3). Of the 3,420 ASVs, 9% co-occurred with tree species, representing 28.2% of the bacterial community abundance, highlighting their significant presence.

While probabilistic co-occurrence analysis offers valuable ecological insights, the analysis of indicator species also contributes significantly to our understanding of ecological dynamics (Barberán et al. 2014). This analysis selects species that are likely to indicate a niche represented by a group (Fig S4). Of the 3,420 ASVs, 15.3% were indicators of tree species, accounting for 38.6% of the bacterial community abundance, also highlighting their significant presence.

**Fig. 4:**
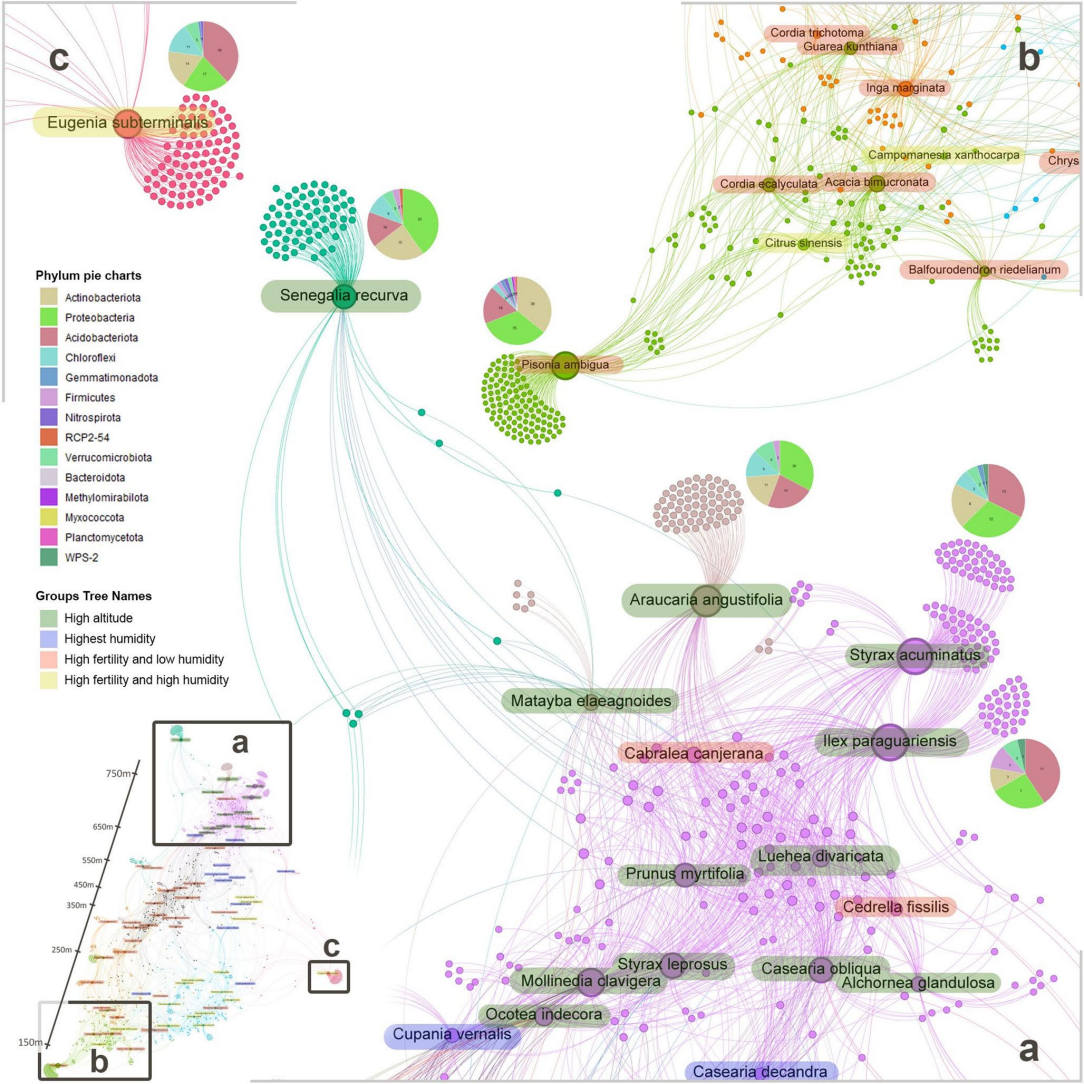
Bacterial taxa correlated with key tree species of the Atlantic Forest. The size of the nodes in the network represents the number of connections that the node has; additionally, the thickness of the line indicates the strength of the correlation. Colors displayed among the nodes and edges followed a statistical community detection based on the Modularity Class (Blondel et al. 2008) for better differentiation of node groups in the outcome. The names of tree species are also highlighted with different colors to differentiate groups of trees at high altitude, high humidity, high fertility and low humidity, and high fertility and high humidity. As the resulting figure was too large, three major areas were magnified. **a** The square at mostly an altitude of 650–750 m was magnified to the lower right corner of the image. **b** The square at an altitude of mostly 150–250 m is shown in the top right corner. **c** *Eugenia subterminalis* with the majority of its presence at 250 m, was positioned at the top left corner of the image. Taxonomy pie charts were positioned next to the ASV clusters of interest. The entire network, shown as a miniature in the bottom left corner of the image, is available in high resolution in the supplementary material (Fig S5)

In addition to probabilistic co-occurrence and indicator species analyses, we conducted a Spearman pairwise correlation analysis (1000 permutations) between each ASV and each tree to further investigate ecological interactions (Fig. 4). Unlike the other methods, which focus on presence-absence patterns or niche indications, Spearman correlation assesses the strength and direction of associations between ASVs and trees based on their rank-order abundance. This approach allows us to identify trends, revealing important correlations between specific ASVs and tree species. These insights enhance our understanding of ecological relationships across gradients, such as environmental conditions or altitudinal variation, providing a valuable complementary perspective to the co-occurrence and indicator species analyses.

Some tree species presented small clusters of ASVs exclusively correlated to them, whereas other species like *Senegalia recurva*,* Araucaria angustifolia*,* Styrax acuminatus*, *Ilex paraguaiensis*,* Eugenia subterminalis*, and *Pisonia ambigua* showed large exclusive ASV clusters in the network (Fig. 4). Of the 1363 correlated ASVs, 749 were correlated with only one specific tree species. The bacteria belonging to the clusters of the key species cited above accounted for 7.44% of the entire community’s abundance. Moreover, *Ilex paraguariensis*, and *Styrax acuminatus* shared a large ASV cluster, which was correlated with these two species. Upon examining the taxonomy assigned to the exclusive ASV clusters, it was found that *Styrax acuminatus*,* Araucaria angustifolia*, and *Ilex paraguaiensis*, were more abundant at higher altitudes, as expected, presented more correlated ASVs belonging to the phylum *Acidobacteriota* (pH ~ 3.9). However, *Senegalia recurva*, despite being more abundant at 550 m and 750 m altitude, presented a lower percentage than expected compared with *Actinobacteriota* and *Proteobacteria* (pH ~ 4.4). Another unexpected finding was the large cluster correlated exclusively with *Eugenia subterminalis*, which despite being a tree almost exclusively present at 250 m altitude, had more correlated *Acidobacteriota* and *Chloroflexi* ASVs, a typical taxonomy composition characteristic of the microbiota at high altitudes. ASVs exclusively correlated to *Pisonia ambigua* had a lower abundance of *Acidobacteriota* and a higher diversity of other taxa (Fig. 4).

In the network, tree species at higher altitudes (650–750 m) show strong correlations with specific ASVs that rarely correlate with tree species at other altitudes (Fig. 4a), while species at lower altitudes (250–350 m) form separate clusters (Fig. 4b). The middle part of the network includes species from 350 m to 650 m, with ASVs correlating across both low and high altitudes.

The Modularity Class applied to the correlation network differentiated tree species across forests types (Fig. 4 and Fig S5). Pink and light pink nodes were predominantly present in the MMOF, whereas light green and black nodes (Fig S5) were mainly present in MSSF and SSSF at altitudes of 450–650 m. Orange and green nodes were primarily present in SSSF and ASSF at 150–450 m. The light blue group (Fig S5) included species present in SSSF and ASSF at 150–450 m with some occurring at higher altitude (550 m). The dark pink group, comprising *Eugenia subterminalis* and *Banara tomentosa* (Fig S5), was strongly present at 250 m of altitude, *with B. tomentosa* also occurring at 750 m altitude. Light gray nodes (Fig S5) represented distributed species across plots.

The colors highlighting the names of tree species in the network (Fig. 4) indicate species of trees separated by Souza et al. (2018) according to similar environmental characteristics. It is possible to observe that the green color represents tree species belonging to the highest altitudes, mostly coinciding with pink and light pink color groups from Modularity Class. Orange-highlighted tree species comprise a set of trees more associated with high fertility soils in low humidity locations, which highly coincide with groups of colors black, green, and orange from Modularity Class (Fig S5). Furthermore, the way the network stabilized after the repulsion of nodes, also separated the high fertility soils in low humidity locations to the left side of the network. On the right side of the network, the trees in blue and yellow, which have high humidity locations, are visible. The tree species in yellow (Fig S5) besides having high humidity, also occur in highly fertile soils.

To evaluate how microbial diversity patterns vary along the altitudinal gradient, we conducted a multivariate analysis of heterogeneity (used here as a proxy for I^2^; Table S4). Ecologically, higher values indicate greater heterogeneity in environmental or compositional conditions among samples within the same altitude class, whereas lower values indicate more homogeneous communities or environmental settings. Heterogeneity showed clear variation across altitudes, with the lowest values observed at 150 m (2.62) and 750 m (3.93), and the highest at 550 m (7.91). Intermediate altitudes (250–450 m) exhibited moderate heterogeneity, ranging from 5.25 to 7.91. The number of bacterial reads correlated with overall tree composition remained consistently high across altitudes, ranging from 51,853 at 350 m to 74,330 at 750 m. In contrast, reads correlated with key tree species varied more widely, with particularly low values at 350–450 m (< 2300 reads) and peaking at 750 m (38,370 reads). Notably, the strongest correlations between microbial and vegetation at 150 m and 750 m coincided with the lowest heterogeneity values (p-value < 0.05; *r* = −0.76). To corroborate to the multivariate dispersion analysis, we also conducted the mean Euclidean distance within the group (Table S4). The same pattern emerged, and the correlation was a bit stronger as in the multivariate measurement (p-value < 0.05; *r* = −0.79).

### Evaluation of the tree influence in the potential ecosystem services of soil bacteria in the Atlantic forest

**Fig. 5:**
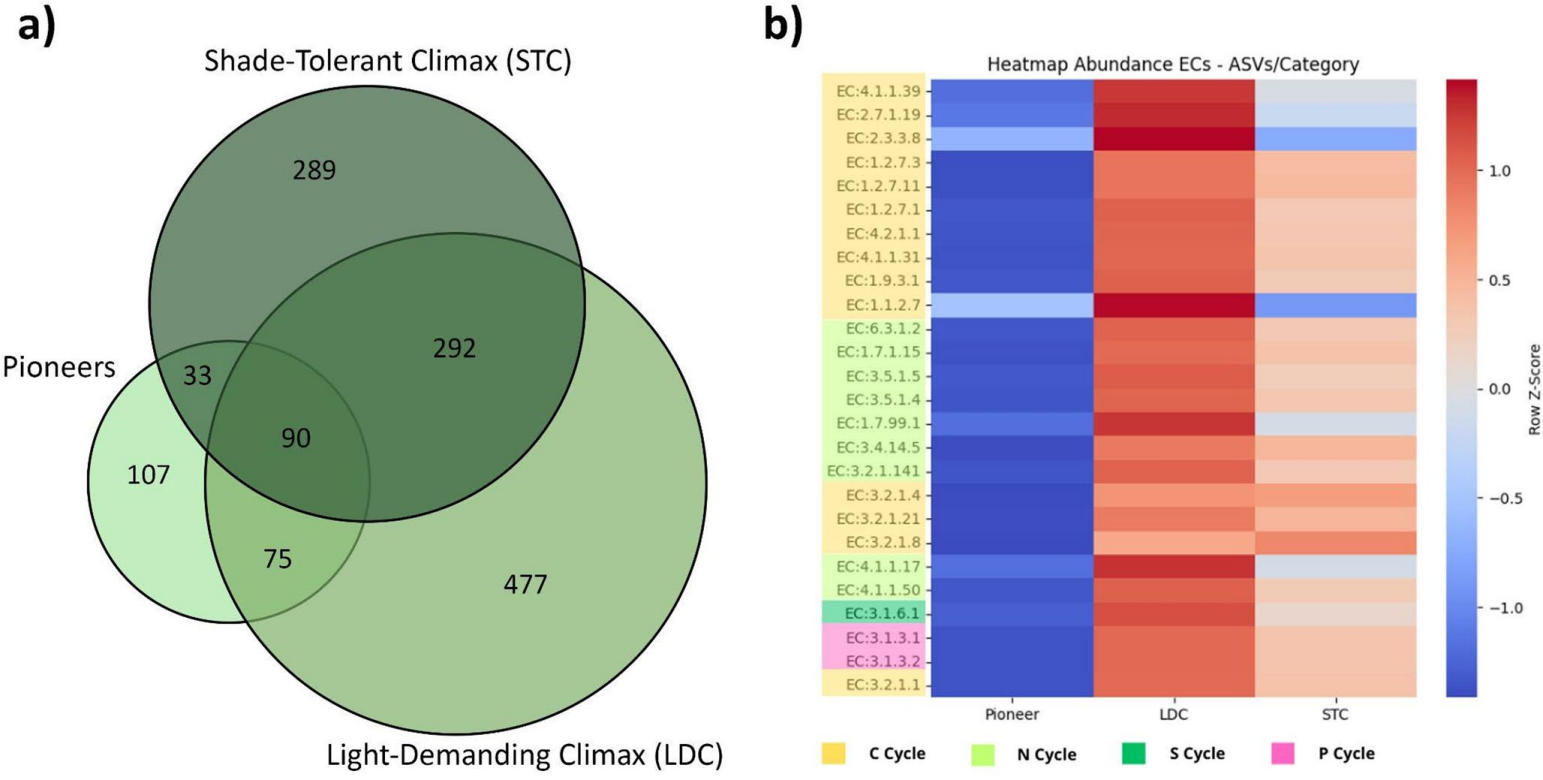
Bacteria correlated with ecological groups of trees and its relation with potential ecosystem services. **a** Euler’s diagram showing the number of ASVs correlated with trees in different ecological groups. **b** Predicted functional potential ecosystem services of ASVs strongly correlated with trees. Heatmap based on the functional metagenomic prediction of ASVs using the PICRUSt2 tool. The metagenome functional potential was predicted using enzyme classification (EC) numbers. The colors on the left of the figure indicate the biogeochemical cycle of the enzymes: *C* cycle in yellow; *N* cycle in green; *S* cycle in dark blue; and *P* cycle in purple

Of the 21 plots, 12 were classified as being in the advanced stage and 9 were considered to be in the intermediate stage of ecological succession (Souza et al. 2015) (Table S3). We analyzed the bacteria that were correlated with the trees from different ecological groups (Pioneers, Shade-Tolerant Climax, Light-Demanding Climax) in the forests of the INP (Fig. 4). Pioneer species are trees that establish rapidly in disturbed or open environments with high light availability and fast growth, dominating early successional stages. Shade-tolerant climax species can germinate and grow under low light beneath a closed canopy and typically dominate mature forests. Light-demanding climax species also occur in late-successional forests but require higher light availability for establishment, often associated with canopy gaps. The numbers of exclusive and shared bacteria by trees in each stage are shown in Fig. 5a.

Pioneer, Light-Demanding Climax (LDC), and Shade-Tolerant Climax (STC) species showed a large number of ASVs shared among these types of trees, suggesting possible microbial inheritance in forest succession (Fig. 5a). To identify the relationship of tree species to the possible soil microbial biogeochemical cycles, the potential ecosystem services performed by ASVs and tree species in Fig. 4 were also analyzed by metagenome functional prediction (Fig S6). An analysis of the predicted functional potential revealed distinct metabolic profiles among the successional groups. Specifically, the Light-Demanding Climax (LDC) group showed a relative increase in the predicted abundance of key enzymes associated with the core pathways of carbon, nitrogen, phosphorus, and sulfur cycling when compared to Pioneer and Shade-Tolerant Climax (STC) groups (Fig. 5b). It is important to note that these results, derived from 16 S rRNA-based predictions (PICRUSt2) and visualized using row-wise Z-score normalization to highlight patterns, indicate modulations in the metabolic potential of the community. They do not confirm active in situ process rates, which are modulated by soil physicochemical conditions. Future studies with metagenomic sequencing and direct enzymatic assays are needed to confirm these functional associations.

## Discussion

The influence of tree species on soil microbial communities is crucial for understanding the relationship between biodiversity and ecosystem function, especially in diverse biomes like tropical forests (Beugnon et al. 2021a; Baldrian et al. 2023). In this study, conducted in the largest protected area of the Atlantic Forest in Brazil, we hypothesized that: (i) based on deterministic processes (niche theory), trees play a crucial role in shaping soil bacterial diversity specifically when abiotic variability is low; and (ii) specific tree taxa in tropical forests function as keystone species, exerting a disproportionate influence on bacterial community structure and function. Our results showed that tree species had a stronger impact on bacterial diversity than soil attributes (Fig. 3), reinforcing the idea that vegetation plays a central role in shaping belowground biodiversity. This finding suggests that plant identity and community composition create unique habitats and microenvironments that select for specific microbial assemblages.

Microbial properties are strongly influenced by vegetation type (Durán and Delgado-Baquerizo 2020) and diversity (Beugnon et al. 2021b; Pei et al. 2016). A positive effect of tree species on microbial communities and their functions has been observed, where tree species richness drives microbial processes, such as respiration, by influencing microbial biomass and diversity (Beugnon et al. 2021a). These effects, noted across biomes, are attributed to increased tree productivity and carbon inputs into the soil, through root exudation (Eisenhauer et al. 2017), and litter production (Huang et al. 2017, 2018). Additionally, tree diversity enhances substrate diversity for soil microorganisms (Eisenhauer et al. 2017; Thoms et al. 2010; Chapman et al. 2013; Eisenhauer et al. 2013). Thus, tree diversity has a dual impact on microbial communities: it increases microbial biomass through higher tree productivity and carbon inputs, while also enhancing the heterogeneity of organic inputs (Hooper et al. 2000). Similar trends were observed in the Atlantic Rainforest, where plant genera were strongly correlated with bacterial and archaeal communities, even within a limited pH range (3.6 to 3.7) (Lima-Perim et al. 2016). In our study, which included a broader pH range (3.5 to 5.6) across 21 plots, tree species remained the dominant factor influencing microbial diversity, underscoring the role of plants in structuring ecological niches. Additionally, tree species, alongside pH and base cations, significantly affected microbial composition in forest soils and litter, highlighting the influence of tree species on nutrient availability and microhabitat conditions (Prescott and Grayston 2013). Our results were also in agreement with the notion that tree species indirectly shape microbial communities by altering environmental conditions (Landesman et al. 2014). While soil pH was an important determinant of microbial variation across forest types, it often reflected the composition of tree species, reinforcing the idea that vegetation shapes microbial distributions. The influence of tree species on microbial abundance and diversity was more pronounced than that of soil properties, impacting both bacterial and fungal communities (Urbanová et al. 2015). Evidence from afforested sites showed that dominant tree species primarily drove soil bacterial community composition, with soil parameters having secondary effects (Liu et al. 2018a). In subtropical mixed forests, tree canopy structure was shown to influence soil C/N ratios, providing an indirect pathway for tree species to shape microbial community structure (Nie et al. 2021).

By mapping interactions between trees and bacterial taxa, we gained insights into how microbial communities depend on forest composition and identified six keystone tree species crucial for maintaining soil bacterial diversity (Fig. 4). *Araucaria angustifolia*, for example, plays a key role in preserving biodiversity in both native and planted areas (Medina et al. 2020), supports subtropical forest regeneration, and provides non-timber products like seeds (Laurans et al. 2012). The dual benefits of *A. angustifolia* for both biodiversity conservation and economic value make it an ideal species for sustainable forest management programs that balance ecological restoration with local community needs. *Styrax acuminatus* is essential for restoring legal reserves and rehabilitating riparian forests, while also supplying wood for cellulose, energy, and resin production (Carvalho 2014). *Ilex paraguariensis* is still harvested from natural environments for tea, though it is increasingly cultivated and managed for conservation, with growing uses in medicine and cosmetics (Dickel et al. 2011). The commercial value of *I. paraguariensis* provides economic incentives for landowners to participate in restoration programs, demonstrating how species selection based on microbial interactions can align conservation goals with economic viability. *Eugenia subterminalis*, a rare endemic species, faces extinction in Paraná due to anthropogenic pressures (CNCFlora, 2012). *Senegalia recurva*, widespread but sensitive to waterlogged soils, is positioned in the network but not closely connected to typical MMOF trees. *Pisonia ambigua* is a well-known tropical and subtropical species, recognized for its growth habit as a woody vine or small tree and its relatively limited distribution (Pramanick et al. 2015). Rather than providing prescriptive guidance, the identification of these keystone species offers a hypothesis-generating basis for evaluating species selection in restoration projects, which should be validated through field-based restoration trials. Dukunde et al. (2019) showed that tree species identity, and to a lesser extent richness, significantly influence bacterial communities. Similarly, our study identified key tree species strongly associated with bacterial diversity and structure.

The probabilistic co-occurrence (Fig. S3), indicator species (Fig. S4), and pairwise correlation analyses (Fig. 4 and Fig S5) revealed gradients related to altitude, forest typology, humidity, and soil fertility. These gradient-specific patterns suggest that restoration strategies should be adapted to local environmental conditions, with different keystone species prioritized depending on altitude and soil characteristics to maximize microbial community benefits. Across all clusters, *Acidobacteriota*, *Actinobacteriota*, and *Proteobacteria* were predominant, which aligns with the patterns observed in soils (Delgado-Baquerizo et al. 2018). *Acidobacteriota* abundance increased in high-altitude soils (700–750 m) with low pH, a trend also observed at lower altitudes in the *Eugenia subterminalis* clusters, where melanic gleysol soils sustain acidic conditions (pH ~ 4.3) (Männistö et al. 2007; Lladó et al. 2017). The cluster of ASVs exclusively correlated with *Pisonia ambigua* exhibited a lower abundance of *Acidobacteriota* and higher taxonomic diversity compared to other clusters (Fig. 4). This finding aligns with Hanif et al. (2019), who noted that *Pisonia ambigua* is more prevalent in low-altitude areas characterized by the diverse environment of an SSSF. Additionally, some ASVs were correlated with tree species at specific altitudes, showing a lower degree of correlation compared to those that were exclusively associated with certain tree species and rarely correlated with species at other altitudes. This suggests that within the networks, microbes are both competitive and complementary, competing for ecological niches that best meet their growth requirements (Faust and Raes 2012). Furthermore, our heterogeneity results (Table S4) show that the stronger microbial associations with tree community and key species at 150 m and 750 m occurred where heterogeneity was lowest, directly supporting our hypothesis that biotic drivers such as vegetation structure influence microbial communities most strongly when abiotic variability is low. This interpretation aligns with findings from Huber et al. (2020), who demonstrated that environmental heterogeneity governs the ecological processes driving bacterial metacommunity assembly. In our case, reduced abiotic variability appears to favor deterministic biotic controls, whereas higher heterogeneity at mid-elevations likely increases the influence of stochastic or environmentally driven processes. From a practical standpoint, these findings suggest that restoration efforts may be most effective when implemented in areas with relatively low environmental heterogeneity, where keystone species can exert maximum influence on soil microbial communities. Together, these patterns underscore the complementary roles of tree diversity and key species in structuring soil bacterial assemblages across environmental gradients.

The presence of shared ASVs among multiple tree species may indicate a complex interplay between tree and bacterial communities, potentially supporting the forest succession process (Zhang et al. 2022a, b). In this study, the forest was in an advanced stage of ecological succession (Table S3), where climax species, both light-demanding and shade-tolerant, are likely inheriting bacterial communities initially associated with pioneer species. Over time, these climax species selectively shape bacterial communities through root exudate-driven recruitment (Cong et al. 2015; Liu et al. 2021). We observed that light-demanding climax species were more strongly correlated with bacterial diversity and predicted metabolic functions related to the sulfur, carbon, phosphorus, and nitrogen cycles (Fig. 5), suggesting that these species may contribute to key microbial biogeochemical processes in the later stages of succession. This microbial inheritance underscores the importance of early ecological groups in shaping long-term microbial and ecological dynamics within forest ecosystems. It also suggests that bacterial communities are not solely influenced by individual tree species, but by broader, community-level interactions that foster ecological resilience and continuity(Van Der Heijden et al. 2007). The shared ASVs among tree species likely reflect a flexible bacterial network, capable of adapting to shifting tree compositions and contributing to the dynamic stability and long-term sustainability of forest ecosystems. This adaptability aligns with the ecological principle of facilitation, where pioneer species from early succession stages support the establishment of later species, fostering a gradual and cooperative transition toward a mature forest state (Connell and Slatyer 1977). Therefore, our results suggest that, given the strong correlation between climax tree species and possible biogeochemical microbial cycles, preserving areas of tropical forests in an advanced stage of ecological succession is crucial for safeguarding their ecosystem services. These findings provide scientific support for current Brazilian forest restoration policies that prioritize areas of high conservation value and suggest that restoration success can be enhanced by incorporating soil microbiome considerations into species selection protocols. An important limitation of this study resides in the predictive nature and limited confidence of marker-based functional analysis. The high NSTI values (Pioneer: ~0.35; LDC: ~0.36; STC: ~0.36) obtained indicate that predictions of enzymatic pathways (ECs) should be viewed with caution. They point to ecological trends and hypotheses to be tested, rather than definitive conclusions about soil functionality. Consistent patterns observed in the heatmap (Fig. 5b), such as the relative modulation of enzymes associated with biogeochemical cycles in the LDC group, remain valid ecological insights but need confirmation by independent techniques, such as shotgun metagenomics or measurements of soil enzyme activities.

## Conclusions

This study underlines the complex interactions between tree species and bacterial communities within the largest protected area of the Atlantic Forest. Based on deterministic processes (niche theory), our findings indicate that tree species strongly influence the abundance of bacteria, an influence that surpasses the impact of soil physico-chemical attributes. Notably, some tree species, like *Senegalia recurva*,* Araucaria angustifolia*,* Styrax acuminatus*,* Ilex paraguaiensis*,* Eugenia subterminalis*, and *Pisonia ambigua*, are identified as the main drivers in these relationships, thus indicating that they might be crucial in the processes of ecosystem maintenance and microbial diversification. The identification of these keystone species offers forest managers practical tools for improving restoration outcomes while aligning with sustainable development goals related to biodiversity conservation and climate change mitigation. Low values of heterogeneity at different altitudes, indicate a higher influence from tree species on the bacterial community. Furthermore, bacteria that are shared among several tree species could reflect complex interactions between tree and bacterial communities and could contribute to the process of forest succession. Given the complexity of biotic relationships in the Brazilian Atlantic Forest, our results underscore the need to conserve and manage tropical forests in advanced stages of ecological succession to preserve biodiversity and ecosystem services, while providing actionable guidance for restoration programs that incorporate soil microbiome considerations into species selection protocols. An interdisciplinary approach is also required for the understanding of ecological interactions that maintain these systems and the resilience mechanisms supporting them. Nonetheless, the relationships identified here are based on observational evidence and should be interpreted as associations, as causal effects cannot be confirmed without experimental manipulation or more rigorous causal-inference approaches.

## Supplementary Information

Below is the link to the electronic supplementary material.

Supplementary Material 1

## Data Availability

Raw sequence data are deposited in the NCBI archive under accession number PRJNA1179706 (https:/www.ncbi.nlm.nih.gov/sra/PRJNA1179706). Furthermore, Supplementary Tables S1 and S2 in the Supplementary Material complement the reproducibility of this work.

## Abbreviations

ASSF: Alluvial seasonal semideciduous forest
ASV: Amplicon sequencing variants
EC: Enzyme classification
INP: Iguaçu national park
LDC: Light-demand climax
MMOF: Montane mixed ombrophylous forest
MSSF: Montane semideciduous seasonal forest
SSSF: Submontane semideciduous seasonal forest
STC: Shade-tolerant climax
