# Supplementary material for: Tree diversity shapes soil bacterial community structure under low abiotic heterogeneity in the Atlantic forest
Source: Arch Microbiol. 2026 Mar 10;208(5):246. doi: 10.1007/s00203-026-04776-y (PMC12975819; doi:10.1007/s00203-026-04776-y)
Supplement: Supplementary file 1 — Supplementary Material 1 [file 203_2026_4776_MOESM1_ESM.docx]

Supplementary Materials for

**Tree Diversity Shapes Soil Bacterial Community Structure under Low Abiotic Heterogeneity in the Atlantic Forest**

D. Tomachewski^1,2^, R. F. D. Souza^4^, D. R. Lammel^2,3^, L. M. Schiebelbein^1^, C. W. Galvão^1^, M. F. Ribeiro^1^, L. P. Karas^1^, F. Galvão^4^, V. A. Baura^5^, M. C. Rillig^2,3^, and R. M. Etto^1^

^1^ Microbial Molecular Biology Laboratory, State University of Ponta Grossa, Ponta Grossa, Brazil

^2^ Freie Universität Berlin, Institut für Biologie, D-14195 Berlin, Germany

^3^ Berlin-Brandenburg Institute of Advanced Biodiversity Research (BBIB), D-14195 Berlin, Germany

^4^ Forest Ecology Laboratory, Federal University of Paraná, Curitiba, Brazil

^5^ Nucleus of Nitrogen Fixation, Federal University of Paraná, Curitiba, Brazil

*Corresponding author: [mazeretto@uepg.br](mailto:mazeretto@uepg.br)

**This PDF file includes:**

Supplementary Fig. 1 to 6

Supplementary Tables 1 to 4


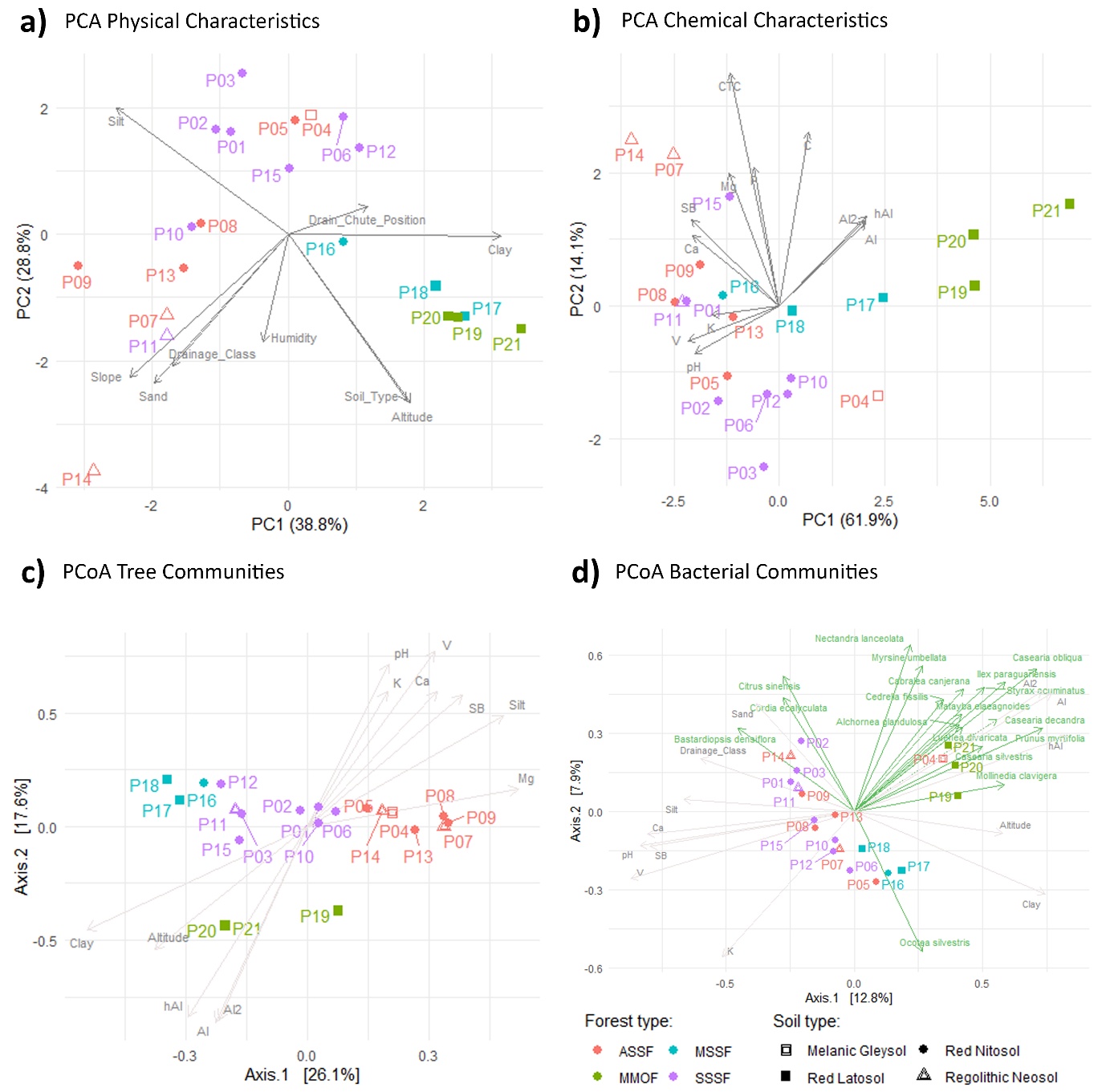


**Fig S1. Main statistical analysis for model creation.**  a) PCA graph of variables for physical characteristics of the plots. b) PCA graph of variables for chemical characteristics of the soil. c) PCoA graph of tree communities. d) PCoA graph of bacterial communities. Physical and chemical characteristics, as well as vegetation, were also computed using *envfit* with 9999 permutations (*p*<0.05). d) PCoA graph of tree communities. Physical and chemical characteristics were also computed using *envfit* with 9999 permutations (*p*<0.05). In the forest types, the acronyms mean: ASSF Alluvial Seasonal Semideciduous Forest, MMOF Montane Mixed Ombrophylous Forest, MSSF Montane Semideciduous Seasonal Forest, SSSF Submontane Semideciduous Seasonal Forest (Souza et al., 2018).


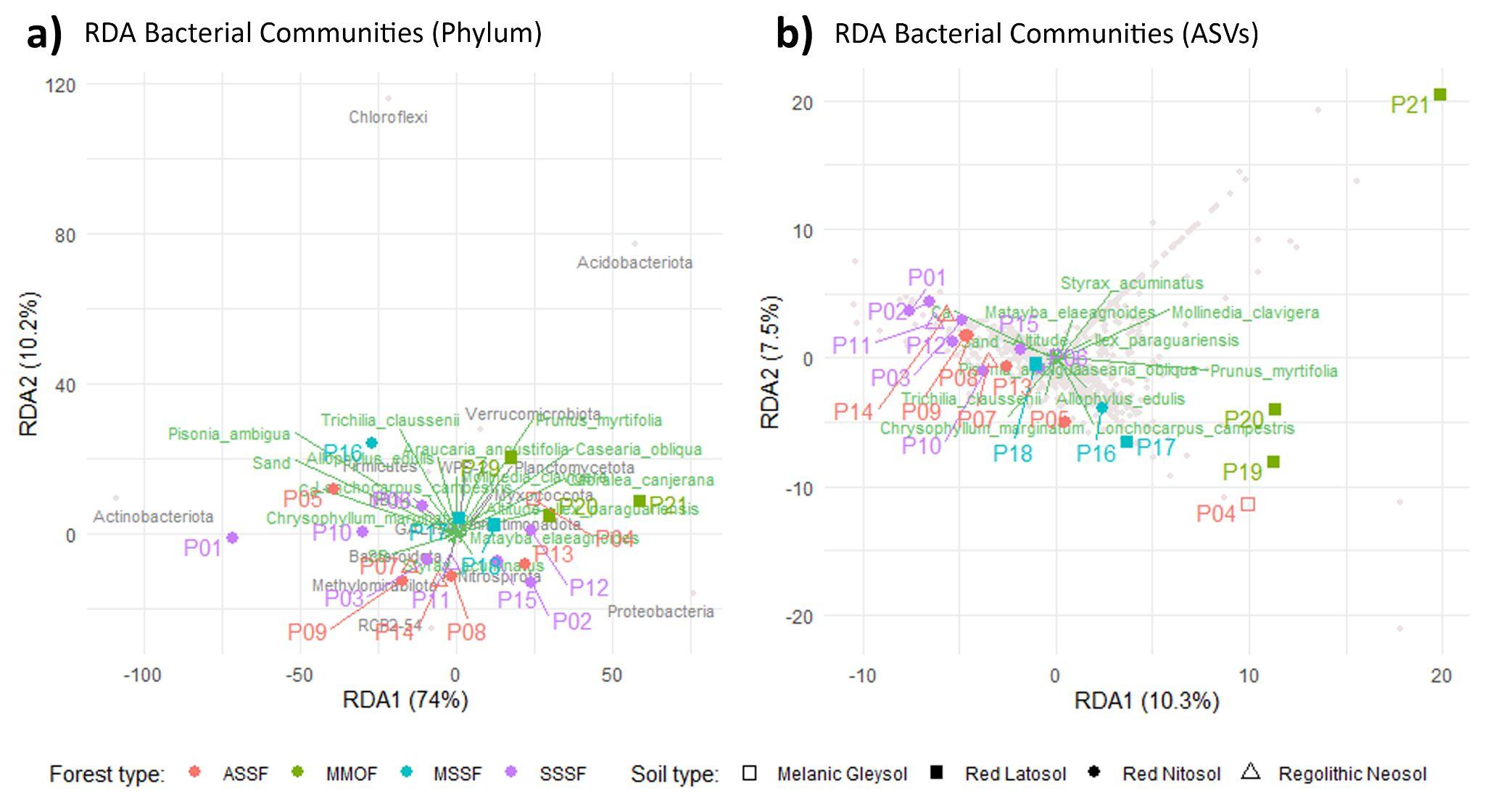


**Fig S2. Main statistical analysis for model creation.**  RDA graph of bacterial communities at phylum (a) taxonomic level and ASVs (b) taxonomic level. Phylum names and points in gray, ASV points in gray, and tree species in dark green. In the forest types, the acronyms mean: ASSF Alluvial Seasonal Semideciduous Forest, MMOF Montane Mixed Ombrophylous Forest, MSSF Montane Semideciduous Seasonal Forest, SSSF Submontane Semideciduous Seasonal Forest (Souza et al., 2018).


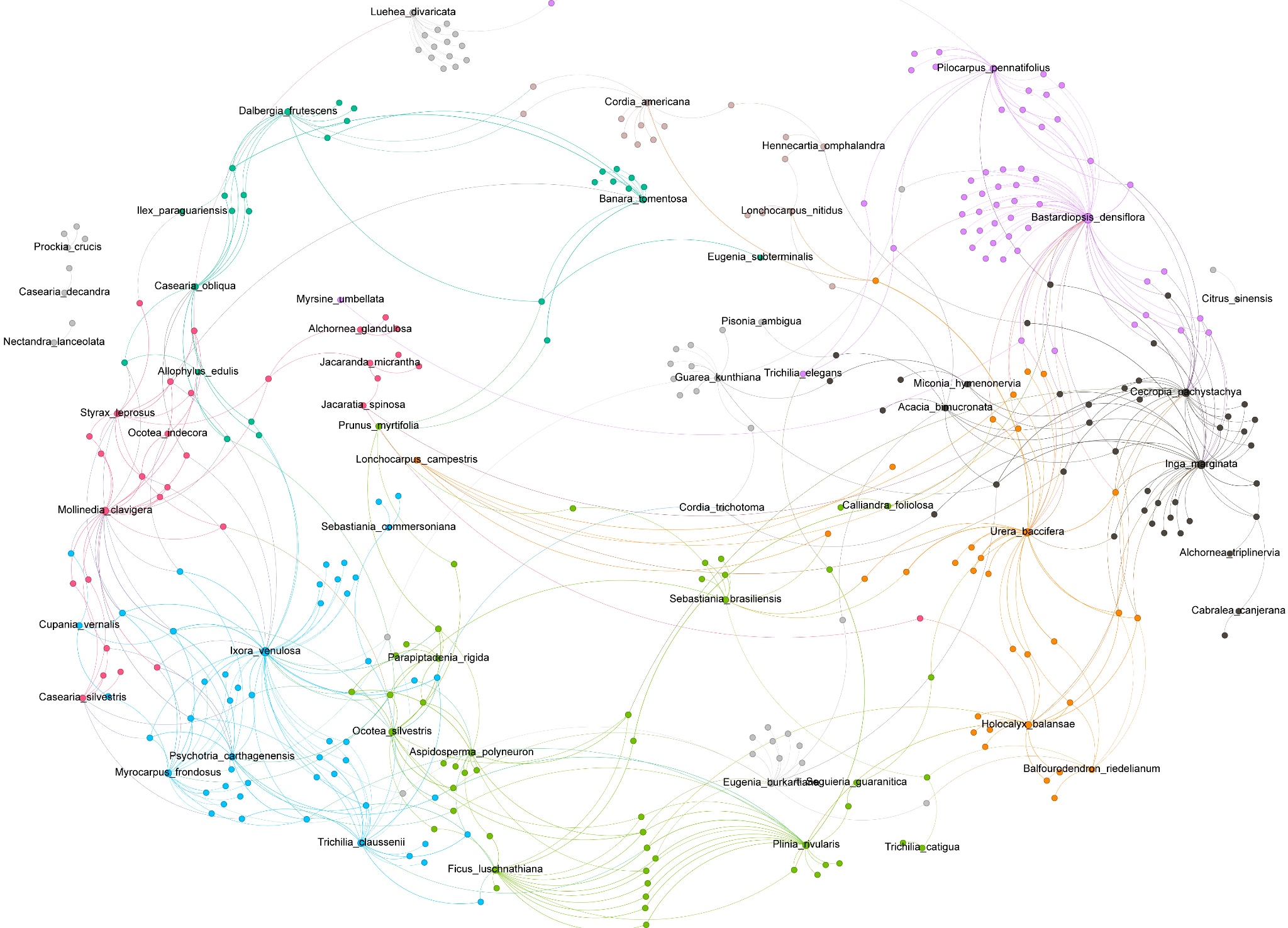


**Fig. S3 Co-occurrence of bacteria and tree species of the Atlantic Forest.** Nodes that represent trees are labeled with the tree name above them, and nodes without names represent co-occurring ASVs. Node and edge colors were assigned using a statistical community detection algorithm based on Modularity Class (Blondel et al., 2008) to highlight distinct node groups in the network. These groups corresponded to different altitudes and forest types, reflecting environmental gradients underlying network structure. From the middle to the left side of the network it can be noticed the tree species most abundant at altitudes of 550 to 750 m shared the co-occurrence with several ASVs, and others have small clusters of ASVs with their own co-occurrence. Attention is drawn to the species *Psychotria carthagenensis*, which has a greater presence at altitudes of 250 m but is closely linked to ASVs co-occurring with trees at higher altitudes. From the middle to the right side to the network, the trees were most abundant at altitudes of 150 to 450 m. These also share ASV co-occurrence with each other and have larger clusters of ASV co-occurrence with specific trees, such as *Bastardiopsis densiflora*. As in the case of *Psychotria carthagenensis*, here *Urera baccifera* (mostly present at 550 m) and *Lonchocarpus campestris* (750 m) were well connected with ASVs co-occurring in low-altitude trees. In addition, the species *Ficus luschnathiana* (most present at 450 m and 650 m) and *Plinia rivularis* (most present at 250 m and 650 m) showed several ASVs co-occurring with each other. A high-resolution image is also available at <https://sites.uepg.br/labmom/public/files/ASVs_trees_cooccur_network>


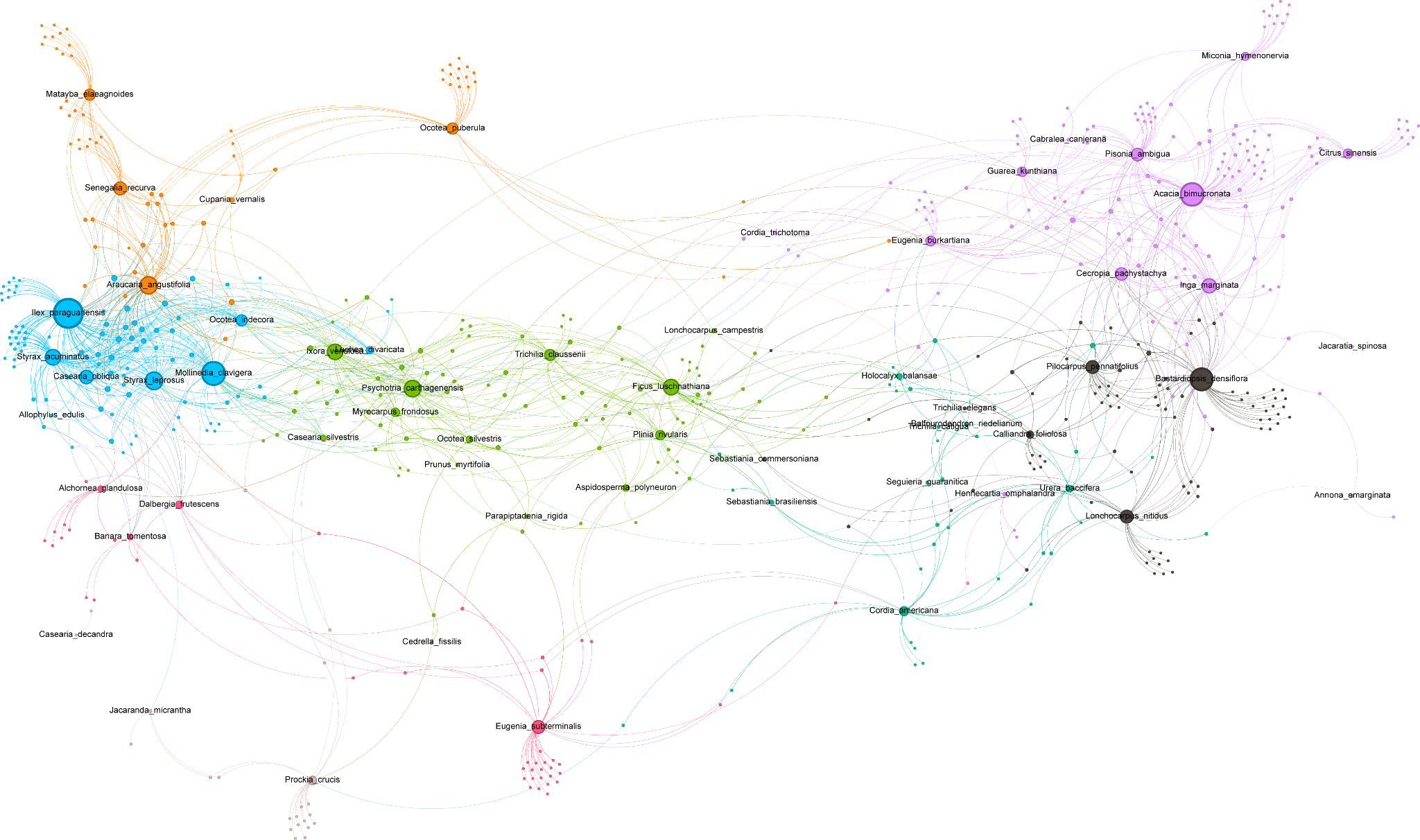


**Fig. S4 Bacteria are shown as indicators of the presence of tree species.** Nodes that represent trees are labeled with the tree name above them, and nodes without names represent ASVs as indicators of tree species. The size of the nodes in the network represents the number of connections that the node has; additionally, the thickness of the line represents how strong the indicator value (Fidelity * Exclusivity) is. Node and edge colors were assigned using a statistical community detection algorithm based on Modularity Class (Blondel et al., 2008) to highlight distinct node groups in the network. These groups corresponded to different altitudes and forest types, reflecting environmental gradients underlying network structure This analysis shows the species of bacteria (ASVs) that indicate the presence of each tree in our plots. After inputting the indicator species into the software to generate the network based on the connections between tree nodes and ASVs, the repulsion algorithm produced a network displaying indicator species for typical low-altitude trees, as well as those found at intermediate and high altitudes. Clusters of indicator bacteria associated with the presence of various tree species from different forest typologies can be observed, such as the light blue and orange bacteria, which indicate trees typical of the Mixed Ombrophylous Forest present in the park. The green and red groups represent trees found at medium mid-range and high altitudes, encompassing many species from Montane Semideciduous Seasonal Forest and some from Mixed Ombrophylous Forest. Meanwhile, the light green, black, and pink trees represent typical species from Alluvial Seasonal Semideciduous Forest tree. It is also noted that there were not many bacterial species acting as indicators for trees in very distinct niches; for example, rarely was an ASV found to indicate both a Mixed Ombrophylous Forest tree and a Submontane Semideciduous Seasonal Forest. In addition to these clusters of bacteria indicating common trees in a specific region, there were also small clusters of bacteria that were exclusive indicators for certain trees, such as *Ilex paraguariensis, Matayba elaeagnoides, Ocotea puberula, Prockia crucis, Eugenia subterminalis, Lonchocarpus nitidus, Pilocarpus pennatifolius, Bastardiopsis densiflora, Acacia bimucronata, Pisonia ambigua, and Miconia hymenonervia*. A high-resolution image is also available at <https://sites.uepg.br/labmom/public/files/ASVs_trees_ind_network>


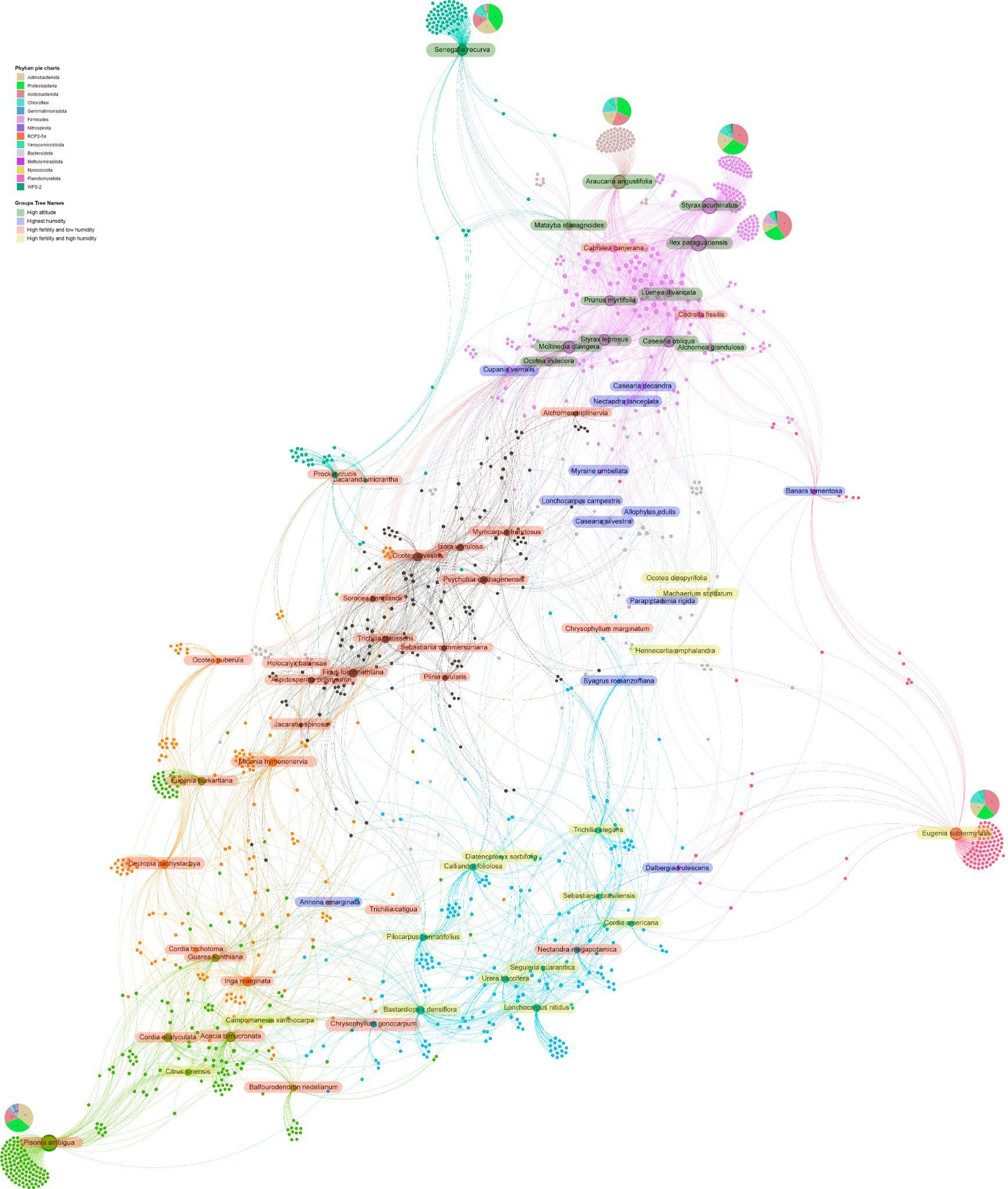


**Fig. S5 Bacterial taxa correlated with key tree species of the Atlantic Forest.** The size of the nodes in the network represents the number of connections that the node has; additionally, the thickness of the line represents how strong the correlation is. Negative correlations were converted to positive to be included in the network, and node and edge colors were assigned using a statistical community detection algorithm based on Modularity Class (Blondel et al., 2008) to highlight distinct node groups in the network. These groups corresponded to different altitudes and forest types, reflecting environmental gradients underlying network structure. Taxonomy pie charts were positioned next to the ASV clusters of interest. A high-resolution image is also available at <https://sites.uepg.br/labmom/public/files/ASVs_trees_corr_network>


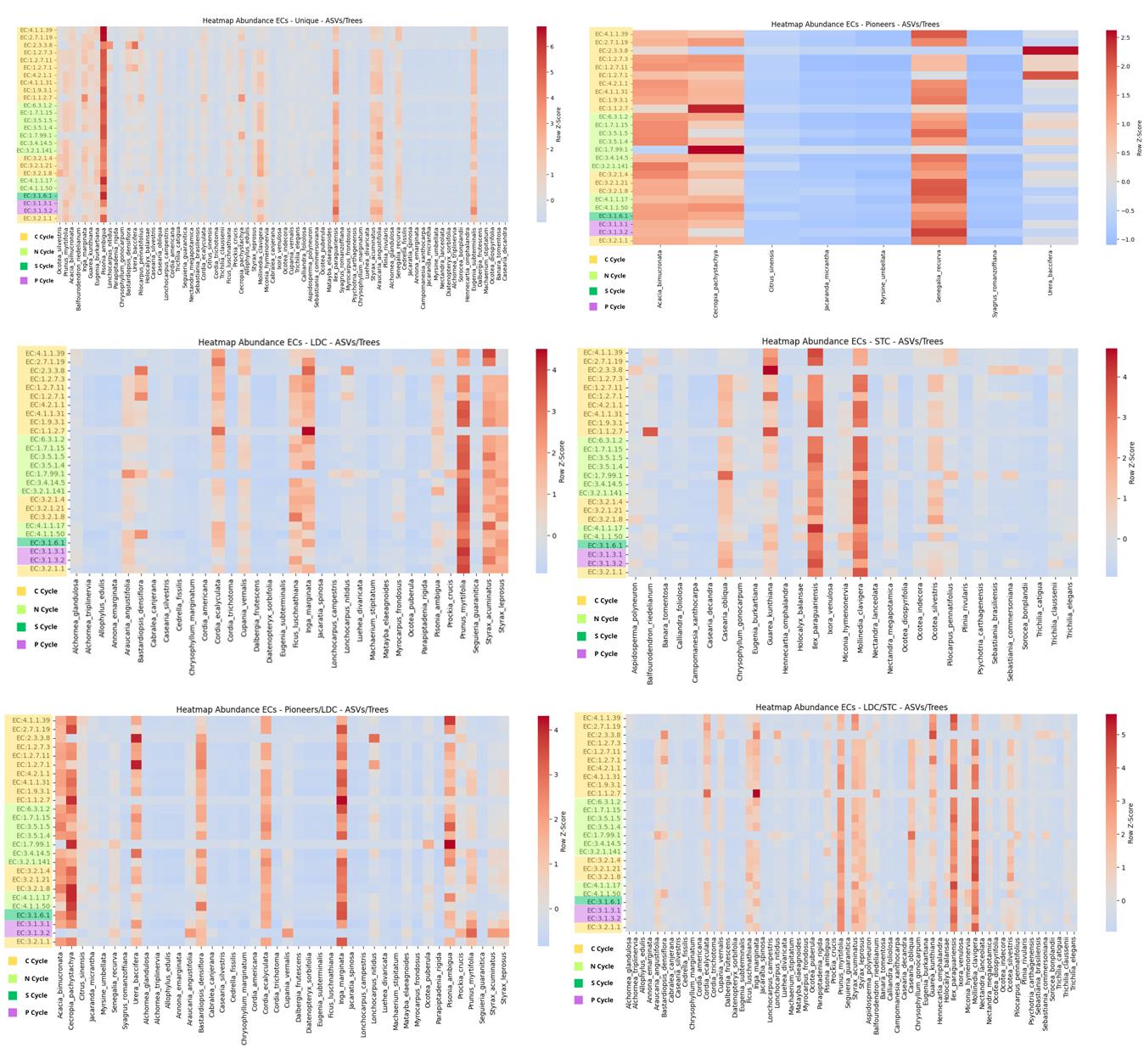


**Fig S6. Heatmap of metagenome function correlated with tree species as predicted by PICRUSt2.**  a) Heatmap of enzyme abundance (ECs) predicted through unique ASVs (1,363 ASVs) associated with different tree species (72 species). b) Heatmap of enzyme abundance (ECs) predicted through ASVs (107 ASVs) associated with pioneer tree species (8 species). c) Heatmap of enzyme abundance (ECs) predicted through ASVs (477 ASVs) associated with Light-Demanding Climax (LDC) tree species (34 species). d) Heatmap of enzyme abundance (ECs) predicted through ASVs (289 ASVs) associated with Shade-Tolerant Climax (STC) tree species (30 species). e) Heatmap of enzyme abundance (ECs) predicted through ASVs (165 ASVs) associated with Pioneer and LDC tree species (42 species). f) Heatmap of enzyme abundance (ECs) predicted through ASVs (382 ASVs) associated with LDC and STC tree species (64 species). All heatmaps are based on the functional metagenomic prediction of ASVs using the PICRUSt2 tool. The color yellow indicates the C Cycle, the color green indicates the N Cycle, the color dark blue indicates the S Cycle, and the color purple indicates the P Cycle.

| Plot | Altitude  (m) | Declivity | Drainage class | pH  CaCl_2_ | P  mg.dm^-3^ | K^+^ | Ca^+2^ | Mg^+2^ | Al^+3^ | H^+^+Al  cmol_c_.dm^-3^ | SB | CTC | V  % | Al  % | Sand  % | Silt  % | Clay  % | Pos. Drainage ramp | Type of Soil | Type of Forest |
| --- | --- | --- | --- | --- | --- | --- | --- | --- | --- | --- | --- | --- | --- | --- | --- | --- | --- | --- | --- | --- |
| P01 | 150 | 2 | 3 | 5.27 | 2.08 | 0.47 | 10.32 | 4.78 | 0.04 | 3.42 | 15.57 | 18.99 | 81.99 | 0.24 | 19.85 | 58.44 | 21.71 | PL | Red Nit. | SSSF |
| P02 | 150 | 2 | 3 | 5.57 | 3.01 | 0.28 | 10.29 | 2.7 | 0.04 | 2.74 | 13.27 | 16.01 | 82.89 | 0.28 | 20.67 | 60.28 | 19.05 | PL | Red Nit. | SSSF |
| P03 | 150 | 2 | 3 | 5.38 | 1.84 | 0.38 | 7.6 | 2.13 | 0.04 | 3.18 | 10.11 | 13.29 | 76.08 | 0.37 | 8.47 | 67.53 | 24 | PL | Red Nit. | SSSF |
| P04 | 250 | 1 | 1 | 4.32 | 2.42 | 0.34 | 4.03 | 2.29 | 0.84 | 5.76 | 6.66 | 12.42 | 53.63 | 11.16 | 12.74 | 61.73 | 25.54 | BA | Mel. Gley. | ASSF |
| P05 | 250 | 2 | 2 | 5.43 | 2.79 | 0.69 | 8.82 | 2.58 | 0.04 | 3.42 | 12.08 | 15.5 | 77.94 | 0.31 | 10.96 | 57.85 | 31.2 | EN INF | Red Nit. | ASSF |
| P06 | 250 | 2 | 3 | 4.9 | 1.98 | 0.82 | 5.12 | 3.59 | 0.19 | 4.28 | 9.53 | 13.81 | 69.01 | 1.93 | 7.55 | 53.38 | 39.07 | PL | Red Nit. | SSSF |
| P07 | 350 | 4 | 4 | 4.76 | 3.67 | 0.45 | 13.75 | 4.6 | 0.04 | 4.28 | 18.8 | 23.08 | 81.46 | 0.2 | 18.88 | 61.22 | 19.9 | EN INF | Reg. Neo. | ASSF |
| P08 | 350 | 3 | 3 | 5.49 | 0.37 | 0.82 | 12.25 | 3.26 | 0.04 | 3.18 | 16.33 | 19.51 | 83.7 | 0.23 | 16.46 | 59.3 | 24.24 | BA | Red Nit. | ASSF |
| P09 | 350 | 4 | 4 | 5.47 | 7.49 | 0.45 | 9.27 | 3.94 | 0.04 | 3.18 | 13.66 | 16.84 | 81.12 | 0.27 | 20.49 | 67.74 | 11.77 | BA | Red Nit. | ASSF |
| P10 | 450 | 4 | 3 | 4.87 | 3.01 | 0.47 | 6.91 | 2.31 | 0.14 | 4.61 | 9.69 | 14.3 | 67.76 | 1.43 | 14.44 | 61.1 | 24.46 | BA | Red Nit. | SSSF |
| P11 | 450 | 4 | 4 | 5.66 | 2.42 | 0.77 | 13.13 | 2.08 | 0.04 | 3.68 | 15.97 | 19.65 | 81.28 | 0.23 | 26.54 | 57.93 | 15.53 | EN SUP | Reg. Neo. | SSSF |
| P12 | 450 | 2 | 3 | 4.94 | 3.01 | 0.65 | 7.31 | 1.62 | 0.17 | 4.96 | 9.58 | 14.54 | 65.89 | 1.74 | 8.6 | 53.11 | 38.28 | PL | Red Nit. | SSSF |
| P13 | 550 | 3 | 3 | 5.07 | 1.32 | 0.63 | 8.34 | 3.96 | 0.1 | 4.61 | 12.93 | 17.54 | 73.72 | 0.79 | 22.36 | 59.85 | 17.79 | BA | Red Nit. | ASSF |
| P14 | 550 | 5 | 5 | 5.85 | 7.64 | 0.72 | 14.47 | 3.6 | 0.04 | 3.42 | 18.78 | 22.2 | 84.6 | 0.2 | 42.24 | 48.46 | 9.3 | EN SUP | Reg. Neo. | ASSF |
| P15 | 550 | 2 | 3 | 4.9 | 4.41 | 0.72 | 9.19 | 3.69 | 0.08 | 3.97 | 13.6 | 17.57 | 77.41 | 0.55 | 15.42 | 59.72 | 24.86 | PL | Red Nit. | SSSF |
| P16 | 650 | 2 | 2 | 5.58 | 2.5 | 0.64 | 9.72 | 2.94 | 0.08 | 4.28 | 13.31 | 17.59 | 75.67 | 0.56 | 17.01 | 48.29 | 34.7 | BA | Red Nit. | MSSF |
| P17 | 650 | 2 | 3 | 4.33 | 4.63 | 0.38 | 3.61 | 2.67 | 0.9 | 7.76 | 6.66 | 14.42 | 46.18 | 11.94 | 15.5 | 38.72 | 45.78 | PL | Red Lat. Di | MSSF |
| P18 | 650 | 2 | 3 | 4.88 | 2.28 | 0.4 | 7.62 | 2.72 | 0.21 | 5.35 | 10.74 | 16.09 | 66.74 | 1.89 | 12.01 | 47.01 | 40.98 | PL | Red Lat. Eu | MSSF |
| P19 | 750 | 2 | 2 | 3.95 | 0.81 | 0.22 | 2.36 | 2.52 | 1.92 | 10.45 | 5.1 | 15.55 | 32.78 | 27.34 | 12.55 | 42.56 | 44.89 | BA | Red Lat. Di | MMOF |
| P20 | 750 | 2 | 3 | 3.97 | 1.4 | 0.14 | 2.44 | 2.92 | 1.96 | 11.26 | 5.49 | 16.75 | 32.78 | 26.35 | 14.82 | 43.82 | 41.36 | PL | Red Lat. Di | MMOF |
| P21 | 750 | 2 | 3 | 3.73 | 3.38 | 0.16 | 1.27 | 1.55 | 3.2 | 13.06 | 2.97 | 16.03 | 18.55 | 51.8 | 12.03 | 34.45 | 53.52 | PL | Red Lat. Di | MMOF |

**Table S1.** **Table with soil attribute data from the plots collected in the INP.** In the position of the drainage ramp, the acronyms are: PL: Plateau, EN INF: Lower Slope, EN SUP: Upper Slope, BA: Base. In the forest types, the acronyms mean: ASSF Alluvial Seasonal Semideciduous Forest, MMOF Montane Mixed Ombrophylous Forest, MSSF Montane Semideciduous Seasonal Forest, SSSF Submontane Semideciduous Seasonal Forest. For the soil types, the acronyms are: Mel. Gley.: Melanic Gleysol, Red Nit.: Red Nitosol, Reg. Neo.: Regolithic Neosol, Red Lat. Di: Red Latosol Dysthrofic, Red Lat. Eu: Red Latosol Euthrophic (Souza et al., 2018).

| **Tree Species** | **P01** | **P02** | **P03** | **P04** | **P05** | **P06** | **P07** | **P08** | **P09** | **P10** | **P11** | **P12** | **P13** | **P14** | **P15** | **P16** | **P17** | **P18** | **P19** | **P20** | **P21** | **EG** |
| --- | --- | --- | --- | --- | --- | --- | --- | --- | --- | --- | --- | --- | --- | --- | --- | --- | --- | --- | --- | --- | --- | --- |
| *Parapiptadenia rigida* | 4 | 0 | 0 | 6 | 4 | 6 | 4 | 2 | 0 | 3 | 0 | 5 | 2 | 0 | 1 | 0 | 1 | 0 | 17 | 0 | 2 | LDC |
| *Cordia americana* | 1 | 0 | 0 | 4 | 6 | 2 | 11 | 2 | 6 | 2 | 0 | 0 | 6 | 1 | 0 | 0 | 0 | 0 | 0 | 0 | 0 | LDC |
| *Acacia bimucronata* | 3 | 1 | 4 | 0 | 0 | 0 | 0 | 0 | 0 | 2 | 0 | 2 | 0 | 0 | 0 | 0 | 0 | 0 | 0 | 0 | 0 | P |
| *Trichilia catigua* | 6 | 12 | 6 | 15 | 4 | 5 | 11 | 1 | 1 | 2 | 3 | 4 | 0 | 0 | 0 | 4 | 4 | 4 | 1 | 0 | 0 | STC |
| *Chrysophyllum gonocarpum* | 14 | 9 | 6 | 10 | 4 | 7 | 4 | 5 | 5 | 4 | 4 | 3 | 13 | 4 | 7 | 1 | 1 | 1 | 4 | 0 | 1 | STC |
| *Allophylus edulis* | 5 | 0 | 0 | 1 | 7 | 2 | 8 | 0 | 1 | 3 | 3 | 0 | 4 | 0 | 0 | 0 | 2 | 0 | 27 | 4 | 2 | LDC |
| *Chrysophyllum marginatum* | 9 | 1 | 3 | 5 | 7 | 4 | 3 | 4 | 2 | 2 | 1 | 0 | 4 | 0 | 1 | 2 | 5 | 4 | 8 | 1 | 1 | LDC |
| *Holocalyx balansae* | 2 | 4 | 2 | 0 | 2 | 4 | 8 | 1 | 0 | 1 | 0 | 1 | 1 | 2 | 2 | 2 | 3 | 7 | 0 | 0 | 0 | STC |
| *Sorocea bonplandii* | 12 | 8 | 20 | 5 | 7 | 11 | 1 | 0 | 0 | 7 | 20 | 17 | 5 | 4 | 19 | 39 | 39 | 32 | 6 | 9 | 19 | STC |
| *Citrus sinensis* | 7 | 10 | 4 | 1 | 0 | 0 | 0 | 0 | 0 | 0 | 0 | 0 | 0 | 0 | 0 | 0 | 0 | 0 | 0 | 0 | 1 | P |
| *Casearia decandra* | 3 | 2 | 0 | 6 | 4 | 1 | 1 | 0 | 1 | 1 | 0 | 0 | 0 | 0 | 2 | 0 | 0 | 0 | 12 | 0 | 8 | STC |
| *Seguieria guaranitica* | 10 | 2 | 0 | 5 | 2 | 1 | 4 | 2 | 0 | 4 | 0 | 0 | 2 | 1 | 0 | 1 | 0 | 1 | 0 | 0 | 0 | LDC |
| *Nectandra megapotamica* | 13 | 5 | 2 | 11 | 7 | 10 | 18 | 16 | 11 | 5 | 3 | 4 | 10 | 13 | 5 | 8 | 2 | 4 | 2 | 1 | 0 | STC |
| *Diatenopteryx sorbifolia* | 3 | 8 | 0 | 4 | 2 | 2 | 13 | 2 | 8 | 1 | 0 | 5 | 2 | 1 | 0 | 2 | 0 | 1 | 4 | 2 | 1 | LDC |
| *Luehea divaricata* | 2 | 0 | 0 | 0 | 0 | 0 | 5 | 4 | 3 | 1 | 0 | 0 | 0 | 0 | 0 | 0 | 0 | 0 | 5 | 8 | 5 | LDC |
| *Balfourodendron riedelianum* | 12 | 10 | 12 | 8 | 10 | 3 | 1 | 13 | 7 | 11 | 0 | 7 | 6 | 8 | 3 | 9 | 8 | 7 | 0 | 0 | 0 | STC |
| *Inga marginata* | 8 | 1 | 2 | 0 | 0 | 0 | 0 | 0 | 0 | 4 | 2 | 0 | 1 | 4 | 1 | 3 | 0 | 0 | 0 | 0 | 0 | LDC |
| *Campomanesia xanthocarpa* | 5 | 10 | 7 | 3 | 3 | 4 | 3 | 2 | 1 | 2 | 1 | 1 | 2 | 0 | 3 | 1 | 1 | 0 | 26 | 1 | 2 | STC |
| *Cedrella fissilis* | 3 | 4 | 6 | 3 | 5 | 4 | 0 | 1 | 0 | 4 | 0 | 2 | 6 | 3 | 7 | 1 | 2 | 0 | 5 | 8 | 10 | LDC |
| *Jacaranda micrantha* | 1 | 0 | 0 | 0 | 0 | 0 | 0 | 1 | 0 | 0 | 0 | 0 | 1 | 2 | 2 | 1 | 1 | 0 | 0 | 1 | 2 | P |
| *Alchornea glandulosa* | 1 | 0 | 0 | 0 | 0 | 3 | 0 | 0 | 0 | 0 | 0 | 0 | 0 | 0 | 6 | 0 | 0 | 1 | 0 | 11 | 8 | LDC |
| *Cordia trichotoma* | 4 | 2 | 0 | 2 | 0 | 2 | 0 | 1 | 0 | 0 | 2 | 2 | 0 | 3 | 1 | 0 | 1 | 0 | 0 | 0 | 2 | LDC |
| *Machaerium stipitatum* | 2 | 10 | 4 | 15 | 11 | 10 | 5 | 9 | 27 | 3 | 9 | 0 | 2 | 5 | 9 | 4 | 0 | 3 | 17 | 0 | 4 | LDC |
| *Cabralea canjerana* | 5 | 10 | 9 | 0 | 2 | 3 | 0 | 2 | 2 | 7 | 7 | 8 | 0 | 7 | 13 | 5 | 6 | 6 | 24 | 19 | 21 | LDC |
| *Ocotea diospyrifolia* | 9 | 9 | 6 | 12 | 10 | 9 | 8 | 25 | 5 | 5 | 4 | 4 | 4 | 4 | 4 | 4 | 4 | 3 | 11 | 4 | 7 | STC |
| *Alchornea triplinervia* | 3 | 3 | 3 | 0 | 0 | 0 | 1 | 1 | 2 | 5 | 6 | 3 | 2 | 3 | 3 | 6 | 14 | 9 | 2 | 14 | 7 | LDC |
| *Miconia hymenonervia* | 2 | 0 | 0 | 0 | 0 | 0 | 0 | 0 | 0 | 3 | 1 | 1 | 0 | 0 | 2 | 0 | 0 | 0 | 0 | 1 | 0 | STC |
| *Hennecartia omphalandra* | 1 | 1 | 0 | 11 | 1 | 0 | 5 | 0 | 0 | 0 | 0 | 0 | 1 | 1 | 1 | 0 | 0 | 0 | 0 | 1 | 0 | STC |
| *Casearia silvestris* | 2 | 2 | 0 | 7 | 0 | 2 | 0 | 0 | 0 | 0 | 1 | 0 | 0 | 0 | 0 | 1 | 1 | 0 | 14 | 1 | 0 | LDC |
| *Cordia ecalyculata* | 3 | 6 | 3 | 1 | 0 | 1 | 0 | 0 | 0 | 1 | 1 | 0 | 1 | 1 | 2 | 0 | 1 | 3 | 1 | 1 | 0 | LDC |
| *Syagrus romanzoffiana* | 1 | 0 | 3 | 3 | 3 | 1 | 16 | 13 | 10 | 5 | 4 | 1 | 5 | 3 | 2 | 1 | 2 | 0 | 12 | 5 | 6 | P |
| *Guarea kunthiana* | 3 | 7 | 33 | 0 | 0 | 0 | 0 | 0 | 0 | 0 | 14 | 11 | 0 | 0 | 0 | 0 | 2 | 10 | 0 | 0 | 0 | STC |
| *Eugenia burkartiana* | 1 | 1 | 0 | 0 | 1 | 0 | 0 | 0 | 0 | 1 | 0 | 4 | 0 | 0 | 0 | 1 | 0 | 1 | 0 | 0 | 0 | STC |
| *Myrsine umbellata* | 1 | 1 | 2 | 5 | 1 | 2 | 3 | 3 | 6 | 2 | 1 | 0 | 4 | 7 | 1 | 0 | 0 | 0 | 5 | 2 | 16 | P |
| *Nectandra lanceolata* | 1 | 6 | 10 | 1 | 0 | 0 | 1 | 2 | 3 | 5 | 2 | 1 | 4 | 3 | 0 | 0 | 1 | 0 | 3 | 9 | 18 | STC |
| *Pisonia ambigua* | 0 | 8 | 1 | 0 | 0 | 0 | 0 | 0 | 0 | 0 | 0 | 0 | 0 | 0 | 0 | 0 | 0 | 1 | 0 | 0 | 0 | LDC |
| *Trichilia elegans* | 0 | 1 | 0 | 1 | 1 | 0 | 16 | 3 | 1 | 0 | 1 | 1 | 6 | 0 | 0 | 2 | 0 | 1 | 0 | 0 | 0 | STC |
| *Bastardiopsis densiflora* | 0 | 1 | 4 | 0 | 0 | 2 | 0 | 1 | 6 | 0 | 1 | 0 | 5 | 5 | 0 | 0 | 0 | 0 | 0 | 0 | 0 | LDC |
| *Pilocarpus pennatifolius* | 0 | 1 | 1 | 0 | 0 | 0 | 2 | 1 | 10 | 0 | 2 | 5 | 0 | 0 | 0 | 0 | 0 | 0 | 0 | 0 | 0 | STC |
| *Jacaratia spinosa* | 0 | 1 | 2 | 1 | 0 | 0 | 0 | 0 | 0 | 0 | 3 | 1 | 1 | 1 | 1 | 2 | 1 | 2 | 0 | 0 | 2 | LDC |
| *Cecropia pachystachya* | 0 | 3 | 2 | 0 | 0 | 8 | 0 | 0 | 0 | 1 | 1 | 1 | 0 | 3 | 1 | 3 | 0 | 0 | 0 | 0 | 0 | P |
| *Annona emarginata* | 0 | 1 | 0 | 1 | 0 | 0 | 0 | 0 | 2 | 3 | 4 | 0 | 3 | 2 | 3 | 0 | 0 | 0 | 2 | 0 | 3 | LDC |
| *Casearia obliqua* | 0 | 1 | 0 | 2 | 1 | 0 | 0 | 0 | 0 | 0 | 0 | 0 | 0 | 0 | 0 | 0 | 0 | 0 | 3 | 6 | 4 | STC |
| *Ocotea puberula* | 0 | 1 | 0 | 0 | 0 | 3 | 0 | 0 | 0 | 0 | 0 | 0 | 0 | 8 | 0 | 0 | 0 | 0 | 2 | 0 | 1 | LDC |
| *Plinia rivularis* | 0 | 4 | 0 | 23 | 6 | 1 | 2 | 0 | 0 | 0 | 0 | 1 | 0 | 0 | 0 | 2 | 4 | 10 | 0 | 0 | 0 | STC |
| *Urera baccifera* | 0 | 0 | 1 | 0 | 3 | 3 | 1 | 16 | 18 | 12 | 5 | 0 | 21 | 29 | 1 | 1 | 0 | 0 | 0 | 0 | 0 | P |
| *Lonchocarpus nitidus* | 0 | 0 | 1 | 0 | 0 | 0 | 2 | 0 | 4 | 1 | 0 | 0 | 3 | 1 | 0 | 0 | 0 | 0 | 0 | 0 | 0 | LDC |
| *Lonchocarpus campestris* | 0 | 0 | 1 | 3 | 0 | 2 | 7 | 1 | 0 | 1 | 1 | 0 | 1 | 0 | 1 | 0 | 3 | 0 | 22 | 0 | 0 | LDC |
| *Sebastiania brasiliensis* | 0 | 0 | 0 | 17 | 11 | 4 | 27 | 15 | 11 | 1 | 3 | 5 | 21 | 17 | 1 | 3 | 3 | 2 | 3 | 0 | 0 | STC |
| *Ixora venulosa* | 0 | 0 | 0 | 8 | 0 | 0 | 0 | 0 | 0 | 0 | 0 | 2 | 0 | 0 | 1 | 10 | 10 | 20 | 1 | 0 | 2 | STC |
| *Dalbergia frutescens* | 0 | 0 | 0 | 2 | 1 | 2 | 0 | 1 | 3 | 0 | 2 | 0 | 9 | 0 | 0 | 0 | 0 | 0 | 4 | 1 | 1 | LDC |
| *Ocotea silvestris* | 0 | 0 | 0 | 2 | 7 | 2 | 0 | 0 | 0 | 1 | 4 | 1 | 0 | 0 | 3 | 13 | 10 | 16 | 1 | 3 | 3 | STC |
| *Trichilia claussenii* | 0 | 0 | 0 | 1 | 0 | 0 | 0 | 0 | 0 | 0 | 3 | 16 | 0 | 0 | 1 | 4 | 7 | 13 | 0 | 0 | 0 | STC |
| *Prunus myrtifolia* | 0 | 0 | 0 | 1 | 1 | 1 | 0 | 0 | 1 | 0 | 0 | 1 | 3 | 1 | 2 | 1 | 2 | 1 | 9 | 8 | 6 | LDC |
| *Banara tomentosa* | 0 | 0 | 0 | 1 | 3 | 3 | 0 | 1 | 1 | 0 | 0 | 0 | 0 | 0 | 0 | 1 | 0 | 0 | 0 | 3 | 1 | STC |
| *Eugenia subterminalis* | 0 | 0 | 0 | 12 | 2 | 3 | 0 | 0 | 0 | 0 | 0 | 0 | 1 | 0 | 0 | 0 | 0 | 0 | 0 | 0 | 0 | LDC |
| *Myrocarpus frondosus* | 0 | 0 | 0 | 1 | 0 | 0 | 0 | 0 | 0 | 0 | 0 | 1 | 12 | 1 | 0 | 3 | 5 | 2 | 2 | 0 | 0 | LDC |
| *Psychotria carthagenensis* | 0 | 0 | 0 | 18 | 0 | 0 | 0 | 0 | 0 | 0 | 0 | 3 | 0 | 0 | 0 | 2 | 1 | 2 | 1 | 0 | 0 | STC |
| *Sebastiania commersoniana* | 0 | 0 | 0 | 1 | 0 | 0 | 3 | 0 | 2 | 0 | 0 | 2 | 2 | 3 | 3 | 1 | 7 | 3 | 0 | 1 | 0 | STC |
| *Aspidosperma polyneuron* | 0 | 0 | 0 | 0 | 0 | 2 | 0 | 0 | 0 | 5 | 7 | 3 | 1 | 0 | 2 | 7 | 1 | 5 | 0 | 0 | 1 | STC |
| *Ficus luschnathiana* | 0 | 0 | 0 | 0 | 0 | 1 | 0 | 0 | 0 | 0 | 1 | 3 | 0 | 0 | 0 | 1 | 1 | 4 | 0 | 0 | 0 | LDC |
| *Styrax leprosus* | 0 | 0 | 0 | 0 | 0 | 0 | 7 | 0 | 0 | 0 | 0 | 0 | 0 | 0 | 0 | 1 | 0 | 1 | 12 | 1 | 2 | LDC |
| *Calliandra foliolosa* | 0 | 0 | 0 | 0 | 0 | 0 | 10 | 0 | 10 | 0 | 6 | 3 | 0 | 1 | 0 | 1 | 0 | 0 | 0 | 0 | 0 | STC |
| *Matayba elaeagnoides* | 0 | 0 | 0 | 0 | 0 | 0 | 1 | 0 | 0 | 0 | 0 | 0 | 1 | 0 | 1 | 0 | 0 | 0 | 3 | 0 | 10 | LDC |
| *Cupania vernalis* | 0 | 0 | 0 | 0 | 0 | 0 | 0 | 3 | 2 | 0 | 0 | 0 | 2 | 2 | 0 | 2 | 3 | 6 | 4 | 0 | 3 | LDC |
| *Prockia crucis* | 0 | 0 | 0 | 0 | 0 | 0 | 0 | 1 | 0 | 2 | 0 | 0 | 0 | 2 | 4 | 0 | 2 | 0 | 3 | 0 | 0 | LDC |
| *Ocotea indecora* | 0 | 0 | 0 | 0 | 0 | 0 | 0 | 0 | 0 | 3 | 0 | 1 | 0 | 0 | 0 | 0 | 0 | 1 | 1 | 1 | 6 | STC |
| *Senegalia recurva* | 0 | 0 | 0 | 0 | 0 | 0 | 0 | 0 | 0 | 0 | 0 | 0 | 4 | 0 | 5 | 0 | 0 | 0 | 1 | 0 | 3 | P |
| *Mollinedia clavigera* | 0 | 0 | 0 | 0 | 0 | 0 | 0 | 0 | 0 | 0 | 0 | 0 | 0 | 0 | 0 | 6 | 3 | 3 | 28 | 1 | 10 | STC |
| *Styrax acuminatus* | 0 | 0 | 0 | 0 | 0 | 0 | 0 | 0 | 0 | 0 | 0 | 0 | 0 | 0 | 0 | 0 | 0 | 0 | 0 | 11 | 14 | LDC |
| *Ilex paraguariensis* | 0 | 0 | 0 | 0 | 0 | 0 | 0 | 0 | 0 | 0 | 0 | 0 | 0 | 0 | 0 | 0 | 0 | 0 | 6 | 18 | 23 | STC |
| *Araucaria angustifolia* | 0 | 0 | 0 | 0 | 0 | 0 | 0 | 0 | 0 | 0 | 0 | 0 | 0 | 0 | 0 | 0 | 0 | 0 | 11 | 0 | 1 | LDC |

**Table S2. Table of tree species along the sample plots in the Iguaçu National Park.** The 72 tree species are described according to Souza et al. (2017). EG = Ecological Group; P = Pionner; LDC = Light-Demanding Climax; STC = Shade-Tolerant Climax.

| **Plot** | **Coverage Value (%)** | | | **Successional Stage** |
| --- | --- | --- | --- | --- |
|  | **Pioneers** | **Light-Demanding Climax** | **Shade-Tolerant Climax** |  |
| 1 | 10.12 | 89.95 | 94.77 | Intermediary |
| 2 | 5.59 | 102.04 | 85.52 | Advanced |
| 3 | 14.36 | 57.69 | 125.83 | Advanced |
| 4 | 10.81 | 74.60 | 114.09 | Advanced |
| 5 | 9.40 | 97.74 | 92.00 | Advanced |
| 6 | 19.37 | 81.90 | 97.87 | Intermediary |
| 7 | 16.47 | 100.45 | 83.09 | Advanced |
| 8 | 34.57 | 76.81 | 88.63 | Intermediary |
| 9 | 25.08 | 106.32 | 68.60 | Intermediary |
| 10 | 18.64 | 82.35 | 99.01 | Advanced |
| 11 | 5.23 | 50.57 | 144.20 | Advanced |
| 12 | 5.01 | 41.78 | 153.21 | Advanced |
| 13 | 32.17 | 79.38 | 82.13 | Intermediary |
| 14 | 41.79 | 90.24 | 67.21 | Intermediary |
| 15 | 11.62 | 77.26 | 110.02 | Advanced |
| 16 | 5.84 | 54.01 | 139.67 | Advanced |
| 17 | 5.58 | 71.83 | 122.58 | Advanced |
| 18 | 0.00 | 51.08 | 147.40 | Advanced |
| 19 | 11.38 | 138.18 | 49.12 | Intermediary |
| 20 | 9.69 | 121.49 | 68.06 | Intermediary |
| 21 | 21.05 | 100.39 | 78.08 | Intermediary |
| **Overall average:** | **14.94** | **83.15** | **100.53** | **Advanced** |

**Table S3. The coverage value for ecological groups and successional stage of the 21 plots installed in the Iguaçu National Park.** Results indicated a good state of conservation of the forests in general. Of the total 21 plots, twelve were classified as being in the advanced stage and nine were considered to be in the intermediate stage of ecological succession; no plot was classified as being in the initial stage.

| **Altitude** | **Heterogeneity** | **Heterogeneity** | **Reads correlated with trees** | **Reads correlated with key species** |
| --- | --- | --- | --- | --- |
|  | **(multivariate analysis)** | **(mean Euclidean distance)** |  |  |
| 150 | 2.63 | 1.62 | 72,508 | 23,185 |
| 250 | 5.50 | 2.35 | 59,425 | 17,139 |
| 350 | 6.56 | 2.56 | 51,653 | 2,117 |
| 450 | 5.78 | 2.4 | 57,562 | 2,252 |
| 550 | 7.91 | 2.81 | 59,346 | 12,930 |
| 650 | 5.25 | 2.29 | 55,838 | 3,690 |
| 750 | 3.93 | 1.98 | 74,330 | 38,370 |

**Table S4. Comparison between the heterogeneity at different altitudes with reads from ASVs correlated with tree species.** Results indicated higher values of reads correlated when heterogeneity values were lower. At altitudes of 150 and 750 meters, bacterial communities were more significantly influenced by tree species.
